# Divergent regulation of auxin responsive genes in root-knot and cyst nematodes feeding sites formed in Arabidopsis

**DOI:** 10.3389/fpls.2023.1024815

**Published:** 2023-02-15

**Authors:** Patricia Abril-Urias, Virginia Ruiz-Ferrer, Javier Cabrera, Rocio Olmo, Ana Cláudia Silva, Fernando Evaristo Díaz-Manzano, Jose Domínguez-Figueroa, Ángela Martínez-Gómez, Almudena Gómez-Rojas, Miguel Ángel Moreno-Risueno, Carmen Fenoll, Carolina Escobar

**Affiliations:** ^1^ Facultad de Ciencias Ambientales y Bioquímica, Universidad de Castilla-La Mancha, Toledo, Spain; ^2^ Centro de Biotecnología y Genómica de Plantas (CBGP), Universidad Politécnica de Madrid and Instituto de Investigación y Tecnología Agraria y Alimentaria-Consejo Superior de Investigaciones Científicas (UPM-INIA/CSIC), Campus de Montegancedo, Madrid, Spain; ^3^ FFoQSI GmbH—Austrian Competence Centre for Feed and Food Quality, Safety and Innovation, Tulln, Austria; ^4^ Unit of Food Microbiology, Institute of Food Safety, Food Technology and Veterinary Public Health, University of Veterinary Medicine, Vienna, Austria; ^5^ Centro Tecnológico Nacional Agroalimentario "Extremadura", Badajoz, Spain; ^6^ Technical University of Madrid, Madrid, Spain; ^7^ International Research Organization for Advanced Science and Technology (IROAST), Kumamoto University, Kumamoto, Japan

**Keywords:** auxin regulated genes, galls, giant cells, lateral root formation, syncytia

## Abstract

Cysts (CNs) and root-knot nematodes (RKNs) induce specialized feeding cells, syncytia, and giant cells (GCs), respectively, within plant roots. The plant tissues around the GCs usually by respond forming a root swelling called a gall that contains the GCs. The ontogenesis of feeding cells is different. GC formation is a process of new organogenesis from vascular cells, which are still not well characterized, that differentiate into GCs. In contrast, syncytia formation involves the fusion of adjacent cells that have already differentiated. Nonetheless, both feeding sites show an auxin maximum pertinent to feeding site formation. However, data on the molecular divergences and similarities between the formation of both feeding sites regarding auxin-responsive genes are still scarce. We studied genes from the auxin transduction pathways that are crucial during gall and lateral root (LR) development in the CN interaction by using promoter-reporter (GUS/LUC)transgenic lines, as well as loss of function lines of Arabidopsis. The promoters *pGATA23* and several deletions of *pmiR390a* were active in syncytia, as were in galls, but *pAHP6* or putative up-stream regulators as *ARF5/7/19* were not active in syncytia. Additionally, none of these genes seemed to play a key role during cyst nematode establishment in Arabidopsis, as the infection rates in loss of function lines did not show significant differences compared to control Col-0 plants. Furthermore, the presence of only canonical AuxRe elements in their proximal promoter regions is highly correlated with their activation in galls/GCs (*AHP6*, *LBD16*), but those promoters active in syncytia (*miR390*, *GATA23*) carry AuxRe overlapping core *cis*-elements for other transcription factor families (i.e., bHLH, bZIP). Strikingly, *in silico* transcriptomic analysis showed very few genes upregulated by auxins common to those induced in GCs and syncytia, despite the high number of upregulated IAA responsive genes in syncytia and galls. The complex regulation of auxin transduction pathways, where different members of the auxin response factor (ARF) family may interact with other factors, and the differences in auxin sensitivity, as indicated by the lower induction of the *DR5* sensor in syncytia than galls, among other factors, may explain the divergent regulation of auxin responsive genes in the two types of nematode feeding sites.

## Introduction

Plant parasitic nematodes cause serious agronomic losses worldwide (Singh et al., 2015). Two of the most economically relevant are the endoparasitic nematodes: cysts (CNs) and root-knot (RKNs) nematodes. Both induce, within plant roots, elaborate feeding cell syncytia for CNs and giant cells (GCs; included in a pseudoorgan called gall) for RKNs, with the aid of a suite of effectors (Vieira and Gleason, 2019; Mitchum and Liu, 2022; Rutter et al., 2022). However, the ontogenesis of syncytia and GCs is strikingly different. GCs are formed from vascular cells, which are still not well characterized but presumably from the pericycle and/or xylem tissues or vascular cambium (Cabrera et al., 2014a; Olmo et al., 2017; Olmo et al., 2020), that undergo repeated mitosis with partial cytokinesis and DNA endoreduplication, forming a multinucleated cell with highly increased volume and a dense cytoplasm (Escobar et al., 2015). On the other hand, CNs select cambial or procambial cells that become the initial syncytial cells. The syncytium, formed by the incorporation of neighboring root cells through local cell wall dissolution, shares some characteristics with GCs, such as endoreduplication and a dense cytosol (Bohlmann, 2015), and overall, both become the only source of nutrients for the nematode’s development.

Auxins are crucial for the morphogenetic events leading to the differentiation of syncytia and GCs (reviewed in Oosterbeek et al., 2021). However, knowledge of the molecular mechanisms underlying the regulation of auxin-responsive genes in GCs or syncytia, as well as their molecular divergences and similarities, is still limited. It is known that an auxin maximum is built in nematode feeding sites (NFSs) with the aid of several mechanisms. One of them encompasses unbalances in auxin transport promoted by differential expression and localization of a specific combination of PIN-FORMED (PIN; efflux auxin carriers) and AUX1/LAX family proteins (influx carriers) in the NFSs. For instance, auxin transport mediated by PIN1 is needed in the initial syncytial cell, whereas PIN3 and PIN4 distribute the accumulated auxin laterally, favoring the radial expansion of the syncytium (Grunewald et al., 2009). Moreover, the effector 19C07 of the CN *Heterodera schachtii* interacts with LAX3, and it was suggested that it increases auxin influx and induces numerous cell wall remodeling enzymes in syncytia (Lee et al., 2011). Auxin import on the basipetal side of the RKN-feeding sites seems to be induced by the concerted action of AUX1, LAX3, and PIN3 (Kyndt et al., 2016). Another putative mechanism that builds the auxin maxima is the injection of auxin-like compounds identified in nematode secretions (De Meutter et al., 2005; Goverse and Bird, 2011), as well as the manipulation of local auxin catabolic and biosynthesis pathways by some effectors in CNs and RKNs such as the chorismate mutase that could directly alter auxin biosynthetic pathways in the plant (reviewed in Oosterbeek et al., 2021; Rutter et al., 2022). Despite the increased knowledge of the differential transcriptomes of CN and RKN-infection sites in Arabidopsis (Puthoff et al., 2003; Jammes et al., 2005; Barcala et al., 2010), some from microdissected or microaspirated feeding cells (Szakasits et al., 2009; Barcala et al., 2010), and the efforts to classify those genes, few studies have focused on their regulation and function in both NFSs. However, several auxin-responsive genes and promoters have been characterized at CN and RKN infection sites. One of the earlier examples was the synthetic promoter DR5, which contained seven canonical AuxRe motifs activated in both CN and RKN NFSs (Aux Re : TGTCTC; Karczmarek et al., 2004; Absmanner et al., 2013; Cabrera et al., 2014a; Olmo et al., 2020). Another is the promoter of an auxin-responsive gene from the Gretchen Hagen 3 family of soybeans (GH3), which also contains AuxRe elements and is activated in GCs (Hutangura et al., 1999). In recent years, key auxin-regulated genes involved in lateral root (LR) formation such as *LBD16*, *miR390*, *AHP6*, and *GATA23* (Okushima et al., 2007; De Rybel et al., 2010; Bishopp et al., 2011; Dastidar et al., 2019); or upstream regulators of the LR auxin signaling cascades, such as *ARF5* and *IAA12*, *14*, *28* (reviewed in Dastidar et al., 2012), have also been described as crucial for gall and/or GCs development and their promoter’s activation were described in detail during the RKN–Arabidopsis interaction (2016; Cabrera et al., 2014a; Olmo et al., 2020). As for the CNs, a comprehensive study of the regulation of 22 out of the 23 auxin response factors (ARFs) family members described in Arabidopsis after *H. schachtii* infection indicated that some members were expressed within the syncytia, but others showed strong signals in neighboring cells (Hewezi et al., 2015). Transduction cascades mediated by ARFs are complex, as they can homodimerize and bind to DNA (Ulmasov et al., 1997; Guilfoyle et al., 1998; Vernoux et al., 2011; Boer et al., 2014), but they can also heterodimerize with other transcription factors. Some of the described interactions are: ARF8 with the basic helix–loop–helix (bHLH) factor BPEp (Varaud et al., 2011); ARF6 with bHLH (PIF4) and BZR1/BES1 (Oh et al., 2014); or ARF6/8 with the MADS factor FUL (Ripoll et al., 2015). Although a direct interaction between bZIP and ARF transcription factors is not yet proven, Arabidopsis bZIP11-related transcription factors mediate auxin response *via* interaction with the chromatin modulator ADA2b, a subunit of a histone acetylation complex (Weiste and Dröge-Laser, 2014), and bZIP and bHLH recognition sites are sometimes part of composite AuxRe elements (Ulmasov et al., 1995; Cherenkov et al., 2018). Additionally, upstream auxin repressors of ARFs (Aux/IAAs), such as IAA14, are crucial for either CN or RKN infection, as dominant-negative mutants that are resistant to degradation mediated by auxin perception result in increased resistance to CNs and RKNs (Grunewald et al., 2008; Olmo et al., 2020).

In this context, this study analyzes the promoter activation and the role in CN infection sites of genes from auxin transduction pathways already shown to be involved in gall and lateral root (LR) development (Cabrera et al., 2014a; Cabrera et al., 2016; Olmo et al., 2020). We discuss their functional and regulatory differences between both RKN and CN infection sites. In addition, we identified a correlation between promoter activity during CN and RKN interaction with host plants and the presence of different combinations of AuxRe, as well as other overlapping *cis*-elements.

## Materials and methods

For simplicity, we used the term “infection site” throughout the text to refer to both the CN and RKN feeding cells as well as the surrounding cells at the infection or establishment site.

### Nematode populations

*Meloidogyne javanica* Treub (1885) population was maintained *in vitro* on cucumber roots (*Cucumis sativus* cv. Hoffmans) grown in Gamborg medium (Gamborg et al., 1968) with 3% sucrose and 0.8% Daishin agar (pH 6.4). To obtain second-stage juveniles (J2s) for *in vitro* infection assays, egg hatching was performed according to Diaz-Manzano et al. (2016). For the *in vitro* propagation of the *H. schachtii* population (Dr. J. Hofmann, BOKU University, Austria), mustard roots (*Sinapsis alba* cv. Albatros) were grown in the same conditions as cucumber roots (above), but at 23°C. Egg hatching was stimulated with sterile 3 mM ZnCl_2_ following the method of Bohlmann and Wieczorek (2015). Both populations were maintained in the dark for multiplication.

### Plant material, growth conditions, and inoculation with J2s

All *Arabidopsis thaliana* seeds were surface sterilized, grown as described by Olmo et al. (2017), and maintained *in vitro* under long-day photoperiod (16 h light/8 h dark) conditions. A list of all the transgenic lines and mutants assessed together with their references is provided in **Supplementary Table 5**. Except where stated, for all transgenic or mutant lines investigated, at least three independent experiments were performed. The number of plants and/or infection sites assessed is indicated in the figure legends.

Lines *pmiR390a::GUS*, *pmiR390a-519::GUS*, *pmiR390a-555::GUS*, and *pmiR390a-555ΔARE::GUS* were selected using kanamycin (Km; 50 µg/ml) as described in Harrison et al. (2006). Kanamycin-resistant plants were transferred to Gamborg B5 plates (Gamborg et al., 1968) with 0.6% Daishin agar (pH 6.4) 7 days post-germination and inoculated with 15 J2s per plant. Early infection stages were considered 3–7 dpi whereas medium-late infection stages were 13–20 dpi. Growth conditions and infection for all other reporter GUS lines were as described in Olmo et al. (2020). The lines *pGATA23::GUS*, *pAHP6::GUS*, and *pmiR390a::GUS* were previously reported as activated in galls (Cabrera et al., 2016; Olmo et al., 2020; more details on the lines are given in **Supplementary Table 5**).

The loss of function lines (*mir390a-2*; *GATA23-RNAi*; *arf7*, *arf19*, *nph4/arf19*; *arf7/arf19*) and the *pARF5::GUS*, *pARF7::GUS*, and *pARF19::GUS* lines were inoculated with 20–30 *H. schachtii* J2s per plant 4 days after germination. All loss of function lines mentioned were previously shown to be crucial during gall formation (Cabrera et al., 2016; Olmo et al., 2020; more details on the lines are given in **Supplementary Table 5**). For *mir390a-2*, *arf7*, *arf19*, *nph4/arf19*, and *arf7/arf19* lines, the males and females were differentiated based on their morphology 13 days post inoculation (approximately in the fourth stage, before reaching the adult stage, when the male leaves the root to mate with the female) following the method of Bohlmann and Wieczorek (2015). The female and syncytia sizes were measured at 19 dpi, also following the method of Bohlmann and Wieczorek (2015). For the *GATA23-RNAi* line, the number of syncytia was assessed as described by Bohlmann and Wieczorek (2015). Additionally, every plant was measured from the base of the stem to the root apex by using a ruler, and the number of syncytia scored per cm of root in each plant was calculated.

### GUS histochemical assay and analysis

Arabidopsis RKN and CN infection sites were hand dissected and incubated in GUS solution as described in Cabrera et al. (2014a), which is basically 5 mM EDTA (pH 8), 0.05% Triton X-100, 0.5 mM K_3_Fe(CN)_6_, 0.5 mM K_4_Fe(CN)_6_, and 1 mg/ml X-GlcA in 50 mM sodium phosphate buffer. For some of the lines with a strong signal, a prefixation step was performed in 0.5% or 2% glutaraldehyde (5 min under moderate vacuum), and samples were washed three times for 5 min in 50 mM sodium phosphate buffer (pH 7.2). The transgenic lines were evaluated for GUS activity at different infection stages, as indicated in the figure’s legends. Galls were photographed under a Nikon SMZ1000 or Olympus SZX16 stereomicroscope (Nikon Corp., Tokyo, Japan; Olympus, Tokyo, Japan) or Nikon Eclipse 90i microscope (Nikon Corp.).

For the semi-quantification of GUS from the CN and RKN feeding sites, the GUS-blue-color intensity from the images acquired under the same conditions was converted into gray values, and the semi-quantification was based on the signal intensity in the saturation channel following the methods described by Beziat et al. (2017) and Olmo et al. (2020). At least three independent experiments were performed per plant line, and the number of plants assessed is indicated in the figure legends.

### Luciferase imaging and expression analysis

The line *DR5::LUC* (Moreno-Risueño et al., 2010) was used to measure differences in the auxin response within the nematode infection sites formed by RKNs and CNs. Infected plants, at 3–4 days post infection, were placed in a new plate and distributed as shown in **Supplementary Figure 4**, sprayed with 1 ml of 2.5 mM potassium luciferine (D-Luciferin potassium salt, Biosynth FL08608, CymitQuimica S.L., Barcelona), and then imaged using an automated chemiluminescence system with a Hamamatsu EMCCD X2 camera. Brightfield and luciferase images were taken using MetaMorph Microscopy Automation Software. Luciferase images were exposed for 30 s to avoid saturation, obtaining a dynamic range of 0–65,536 levels in a 16-bit image. Images were exported as multidimensional TIF files, and expression was measured by selecting the region of interest (ROI; RKN and CN infection sites, all shown in yellow and red, respectively, in **Supplementary Figure 4**) in ImageJ software (Schneider et al., 2012). Quantifications were expressed as analog digital units (ADUs) per minute. The average mean was defined as the luminescence of the ROI normalized by the area of the ROI; the maximum value of the ROIs was also measured. Data are presented as a percentage taking galls as a reference. Two independent experiments were performed per plant line, and the number of galls and CN infection sites assessed is indicated in the corresponding figure legend.

### Pharmacological treatments

Treatments with 𝛼-(phenyl ethyl-2-one)-indole-3-acetic acid (PEO-IAA), an auxin antagonist that inhibits the auxin signalling pathway by binding to the SCFTIR1/AFBs ubiquitin–ligase complex (Hayashi et al., 2008) were performed as follows: infected plants of the reporter lines indicated in the figures were incubated for 4 days on medium containing either 300 μM PEO-IAA diluted in dimethyl sulfoxide (DMSO) or only in DMSO as a control as described by Olmo et al. (2019; 2020). GUS expression was examined 4 days after treatment. At least three independent experiments were performed, and the number of plants assessed is indicated in the figure legends.

### *In silico* analysis of *cis* elements and transcriptional patterns

The 800 nucleotide sequences upstream of the transcription start sites of the promoter regions of *LBD16*, *AHP6*, *GATA23*, and *miR390* were obtained from the Arabidopsis Information Resource (TAIR) (https://www.arabidopsis.org/index.jsp). The *cis* elements listed in **Supplementary Table 1**, originally described in Dastidar et al. (2019) and Cherenkov et al. (2018), were identified within the gene promoter regions, classified in **Supplementary Table 1**, and represented in **Supplementary Figure 2**. The identification of genes encoding ARFs, Basic Leucine Zipper Domain (bZIP), and Basic Helix–Loop–Helix (bHLH) family members, upregulated either in Arabidopsis galls/GCs or syncytia (**Supplementary Table 2**), was performed from the lists available in NEMATIC (Cabrera et al., 2014b). Detailed information about their expression patterns in galls and syncytia transcriptomes, descriptions, etc. is provided in **Supplementary Table 2**.

## Results

### Activation patterns of main auxin-responsive gene regulators for gall and lateral root formation during the cyst-nematode interaction in Arabidopsis

**Figure 1:**
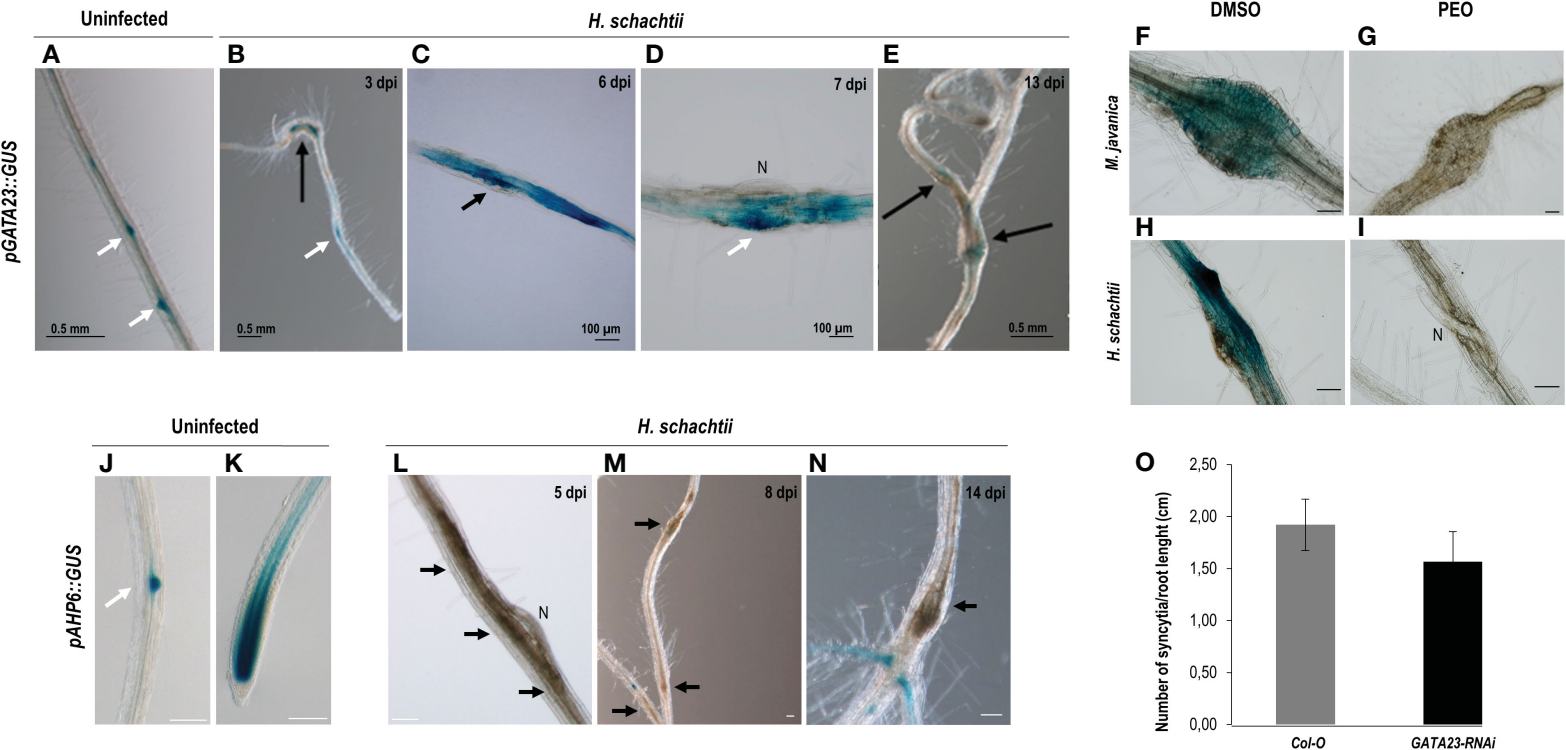
Activation patterns of *pGATA23::GUS* and *pAHP6::GUS* in the infection sites induced by *Heterodera schachtii* in Arabidopsis. The expression in uninfected tissues, used as positive control was centred in the lateral root primordia **(A)**. GUS staining of *pGATA23::GUS* Arabidopsis roots infected by *H*. *schachtii* within the infection sites at early infection stages (**B–D**; 3–7 days post inoculation; dpi), that decreased at 13 dpi **(E)**. GUS staining of RKN and CN infection sites at 7 dpi of the *pGATA23::GUS* line untreated and treated with PEO-IAA (IAA transduction inhibitor; **F–H**, and **G–I**; respectively). An RNA interference line of GATA23, *GATA23-RNAi*, showed no differences in the establishment of **(H)** *schachtii* compared to the control (Student’s t-test, p <0.05) **(O)**. No GUS staining was detected in the *pAHP6::GUS* line at early (5–8 dpi; **L, M**), or medium-late stages of syncytia development (14 dpi; **N**). GUS signal was detected in the positive control, uninfected tissue at lateral root primordia and root tip (**J, K**, respectively) as previously described (Moreira et al., 2010). Black arrows indicate the presence of the syncytia. Scale bars: 100 µm **(F–N)**, white arrows, lateral root primordia, N, nematode. At least 50 independent plants per line and infection time were assayed for GUS analysis, as well as for the evaluation of the infection parameters of *GATA23-RNAi* and its corresponding Col-O control line. At least three independent experiments per independent plant line were performed.

The *GATA23* encodes a transcription factor involved in LR founder cell specification and was also described as crucial for gall formation (Olmo et al., 2020). Here we show that the promoter of *GATA23* was also active at early stages of infection with the CN *H. schachtii* (3–7 dpi; **Figures 1B–D**), and a lower signal was detected at medium-late stages (13 dpi; **Figure 1E**). The signal was centered in the syncytia (**Figures 1C, D**), and some of the LR primordia within them also showed an intense GUS signal (**Figure 1D**; white arrow), similar to the LR primordia in the uninfected roots (**Figure 1A**; see white arrows). The promoter of *GATA23* was activated by auxins in both CN and RKN infection sites, as a treatment with an antagonist of IAA that inhibits the auxin signaling pathway (PEO-IAA) abolished the *pGATA23::GUS* activation in both nematode infection sites (**Figures 1G, I**) compared to the corresponding controls with no inhibitor (**Figures 1F, H**). Additionally, we evaluated the putative function of GATA23 in the cyst nematode–Arabidopsis interaction by using the partial loss of function line, *GATA23-RNAi* (De Rybel et al., 2010). The *GATA23-RNAi* line showed significant differences in the number of syncytia per plant when compared to the control line (**Supplementary Figure 1B**). However, as the transgenic line showed shorter roots than the Col-0 control (p <0.05; **Supplementary Figure 1A**), we normalized the number of syncytia to the root’s length. The results indicated no significant differences in the number of syncytia between the transgenic line and the Col-0 control when normalized per root length (**Figure 1O**). Hence, the role of GATA23 seems to be less crucial for the CN’s establishment than for gall formation. Interestingly, A*HP6*, also induced by an auxin maximum during LR formation (**Figures 1J, K**, Bishopp et al., 2011), was not active in CN-feeding sites in any of the stages of infection assessed, either early (5–8 dpi) or medium-late (14 dpi) (**Figures 1L, M, N**, respectively). However, it was induced in the uninfected controls in the root tip and in LR primordia (**Figures 1J, K**; Moreira et al., 2013).

**Figure 2:**
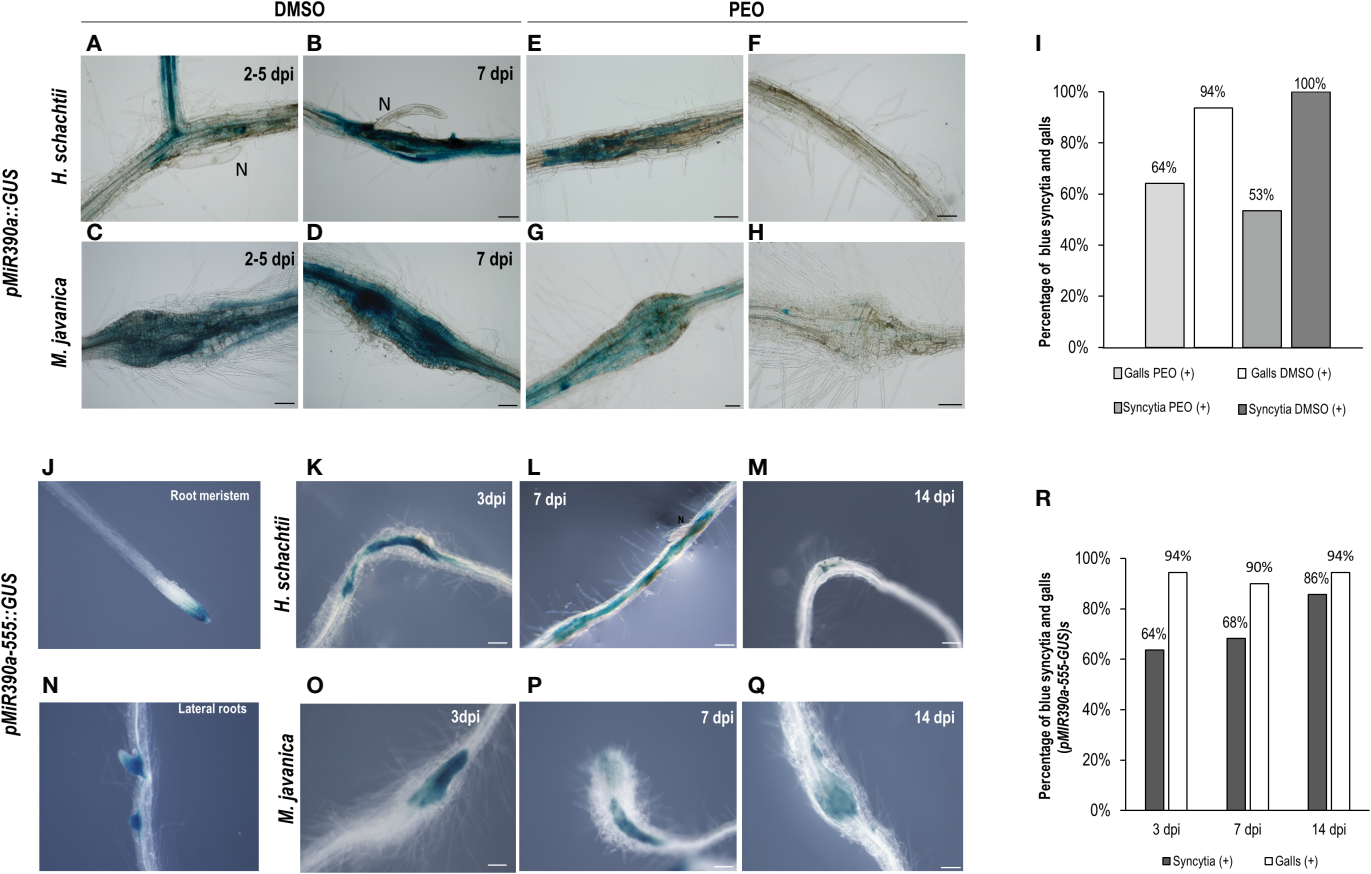
*pmiR390a::GUS* and *pmiR390a-555::GUS* are active in the infection sites induced by *Heterodera schachtii*, in galls induced by *Meloidogyne javanica* in Arabidopsis and partially regulated by auxins. Arabidopsis roots of *pmiR390a::GUS* infected by *H. schachtii or M. javanica* with a strong GUS signal at 2–5 and 7 days post inoculation (dpi; **A–D, **respectively*).* Treatment with the PEO-IAA (IAA transduction inhibitor) inhibited partially its expression (CNs, **B–F** and RKNs infection sites, **D–H**). Percentage of GUS-stained galls and syncytia (7 dpi) in PEO-IAA treatment respect to the DMSO control **(I)**. Activation pattern of a deleted version of the promoter *pMIR390a-555::GUS* in syncytia and galls at 3, 7, and 14 dpi (**K–M** and **O–Q**, respectively) and percentages of GUS-stained RKN and CN infection sites **(R)**. The expression in uninfected tissues was centred in the root meristem and lateral root primordia, positive controls, as described (**J, N**; Dastidar et al., 2019). Scale bars: 100 µm. At least 13 *pmiR390a::GUS* independent plants per treatment and 16 *pMIR390a-555::GUS* plants were assayed by GUS staining. Chi-square analysis, [χ² (2, 51) = 1.2] and [χ² (2, 84) = 0.57], indicated that the distribution of GUS-stained CN and RKN infection sites in the *pMIR390a-555::GUS* line is not significantly different among the three infection stages, P <0.05. Three independent experiments per line assayed were performed.

**Figure 3:**
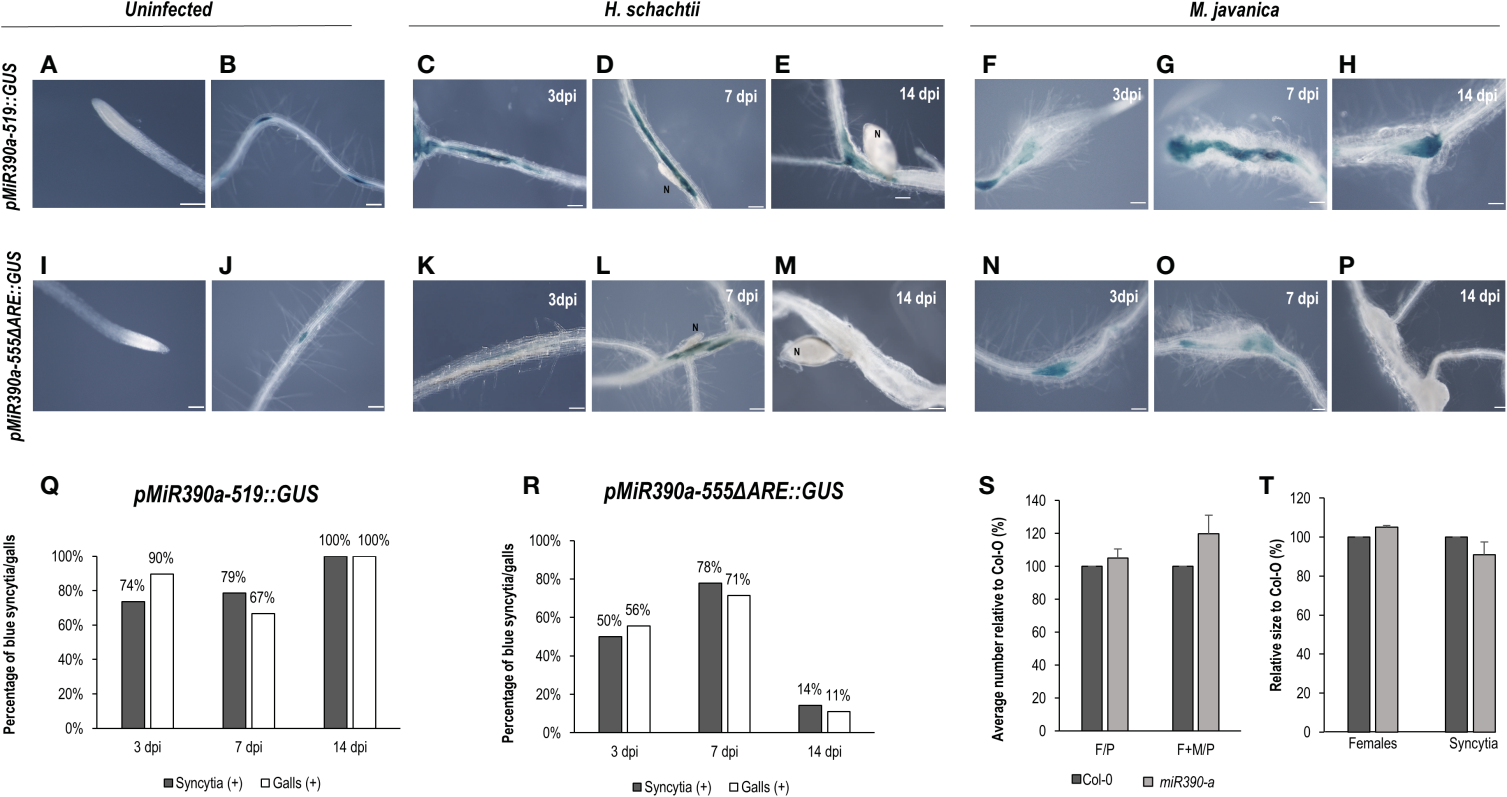
*pmiR390a-519::GUS* and *pmiR390a-555ΔARE::GUS* are active in the infection sites of *Heterodera schachtii* and *Meloidogyne javanica* in Arabidopsis The expression in uninfected tissues was centred in the lateral root primordia in both promoters *pmiR390a-555, pmiR390a-555ΔARE::GUS* **(B, J)**, but suppressed in the root meristem, used as positive controls (**A, I**; Dastidar et al., 2019). GUS-staining of Arabidopsis roots of the *pmiR390a-519::GUS* line infected by *H. schachtii or M. javanica* at 3, 7, and 14 days post inoculation (dpi; **C–H**, respectively). A deleted promoter version of an *AuxRe* element present in *pmiR390a-555, pmiR390a-555ΔARE::GUS*, showed GUS signal at 3, 7 dpi in both infection sites (**K, L, N–O**, respectively), but it was nearly absent at 14 dpi (**M, P**, respectively). Percentage of syncytia and galls with GUS-staining at 3, 7 and 14 dpi in *pmiR390a-519::GUS* **(Q)** and in *pmiR390a-555ΔARE::GUS* **(R)**. A loss of function line *miR390a-2* showed no significant differences in either the establishment of *H. schachtii* **(S)** or the female and syncytia size **(T)** as compared to the control Col-O (Student’s t-test; p <0.05). Number of females measured in Col-0, n = 20, in *miR390a-2*, n = 23; number of syncytia measured, in Col-0, n = 18; in *miR390a-2*, n = 19. Scale bars: 100 µm. At least 18 independent plants were assayed for GUS in each line and at least n ≥40 per line for the infection parameters of the *miR390a-2* line and its corresponding control Col-0. Chi-square analysis, [χ² (2, 47) = 4.22] and [χ² (2, 41) = 3.43], indicated that the distribution of GUS-stained CN and RKN infection sites, respectively, in the *pMIR390a-519::GUS* line was not significantly different among the three infection stages, P <0.05. Chi-square analysis, [χ² (2, 20) = 6.34] and [χ² (2, 25) = 6.57], indicated that the distribution of GUS-stained CN and RKN infection sites, respectively in the *pMIR390a-519::GUS* line was significantly different among the three infection stages, P <0.05. At least three independent experiments per line were performed.

Another gene, also expressed during LR development, corresponds to a microRNA (*miR390*) with a crucial function regulating the biogenesis of tasiRNAs as well as the expression of ARF3 in LRs and galls (Marin et al., 2010; Cabrera et al., 2016). The promoter GUS fusion line, *pmiRNA390a::GUS*, corresponding to the 2.6 kb regulatory region of *MIR390a* (Marin et al., 2010), showed a clear and strong GUS signal within the CNs infection sites at early infection stages (2–5, 7 dpi; **Figures 2A, B**), similar to galls (**Figures 2C, D**). Treatments with the auxin response inhibitor PEO-IAA during gall and syncytia formation indicated that the promoter of *miR390* was partially regulated by auxins in both infection sites, as the proportion of syncytia and galls with positive GUS staining was reduced in the PEO-IAA treatments compared respect to the DMSO control (from 100% to 53% in syncytia and from 94% to 64% within galls; **Figures 2B–I**), but it was not suppressed, as was the case for the *GATA23* promoter after the same treatment (**Figures 1G, I**). It has been described previously that a 555 bp deletion line of the *pmiRNA390a* promoter (*pmiR390a-555::GUS*) containing an auxin-like element (AuxRe) maintained the same activation pattern as the full promoter in LRs and in the root meristem, but a further deletion (519 bp of the promoter region; *pmiR390a-519::GUS*) lacking the AuxRe abolished promoter activation in the root meristem (Dastidar et al., 2019). We obtained the same results when similar systems were used as positive controls (**Figures 2J, N**, **3A)**. Hence, we investigated both constructs after nematode infection. The results indicated that *pmiR390a-555::GUS* containing the AuxRe showed a similar expression pattern as the full promoter (2.6 kb; **Figures 2A–D**) in both nematode infection sites, at early (3 dpi) and medium-late infection stages (7–14 dpi) (**Figures 2K–M, O–Q**). The number of GUS-stained CNs infection sites increased at medium-late infection stages, up to 86% at 14 dpi (**Figure 2R**) and the number of GUS-stained galls was high at all infection stages (up to 94% at 3 and 14 dpi; **Figure 2R**). The line with the 519 deletion from the full promoter, *pmiR390a-519::GUS*, showed a similar induction pattern in both infection sites, with similar activation patterns to that of the 555 bp deletion (**Figures 3C–H**; **Supplementary Table 6**), although the percentage of GUS-stained RKNs and CNs infection sites was slightly lower than in the 555 bp deletion at early infection stages (up to 79% in syncytia and 90% in galls; **Figure 3Q**). Differences in the number of GUS-stained RKN or CN infection sites among the different infection stages assessed either in the *pmiR390a-519::GUS* or in the *pmiR390a-555::GUS* lines were not significant (χ²; p <0.05; see **Figures 2, 3** legends). These results indicated that the 36-bp sequence located between positions −555 and −519 was not necessary for the expression of the reporter GUS gene in both infection sites, similarly to LRs, however it was clearly required for its expression in the primary root meristem (**Figures 3A, B**). Within the 36 bp located between the 555 and the 519 promoter regions, a single copy of an auxin responsive element (AuxRe) was identified as being involved in the transcriptional regulation of *MIR390a via* its interaction with ARF5/MP in the root meristem (Dastidar et al., 2019). Yet, we investigated a version of the *pmiR390a-555::GUS* line with a deletion in this AuxRe element, *pmiR390a-555ΔARE::GUS*. (GGTCTTCGGCCGACAAAAAAAA (WT), GGTCTTCGGC—–AAAAAAA (−555ΔARE); Dastidar et al., 2019) after nematode infection. The results indicated that the GUS signal was maintained in *pmiR390a-555ΔARE::GUS* at early infection stages in CNs and RKNs-infection sites (3, 7 dpi; **Figures 3K–L, N–O**); however, the signal was hardly detected at 14 dpi in both nematode infection sites (**Figures 3M, P**). Concurrently, the percentage of both GUS-stained nematode infection sites at 14 dpi was significantly lower than at earlier infection stages (χ²; p <0.05; see **Figure 3** legend). In addition, significant differences were observed between the activation pattern of the *pmiR390a-555ΔARE::GUS* line and that of the *pmiR390a-555::GUS* and *pmiR390a-519::GUS* lines after CN and RKN infection (p <0.05; **Supplementary Table 6** compare **Figure 2R** to **Figures 3Q, R**). Thus, the deletion of the AuxRe element produced a reduction in the percentage of GUS-stained RKN and CN infection sites, although the signal was not totally suppressed; this agrees with the partial regulation by auxins observed in the longest promoter region assessed, *pmiR390a::GUS*, when treated with PEO-IAA (**Figure 2**). Hence, we investigated whether the loss of function in line *mir390a-2* (**Supplementary Table 5**) may have an impact during CN infection, and no significant differences were observed in the percentage of females or females + males per plant as compared to Col-0 after *H. schachtii* infection (p <0.05; **Figure 3S**). Accordingly, no differences were detected in the size of the females or the syncytia relative to the control line (**Figure 3T**). Therefore, the promoter of *miR390a* was activated, but its corresponding *mRNA390a* did not seem to play a major role during cyst nematode infection.

**Figure 4:**
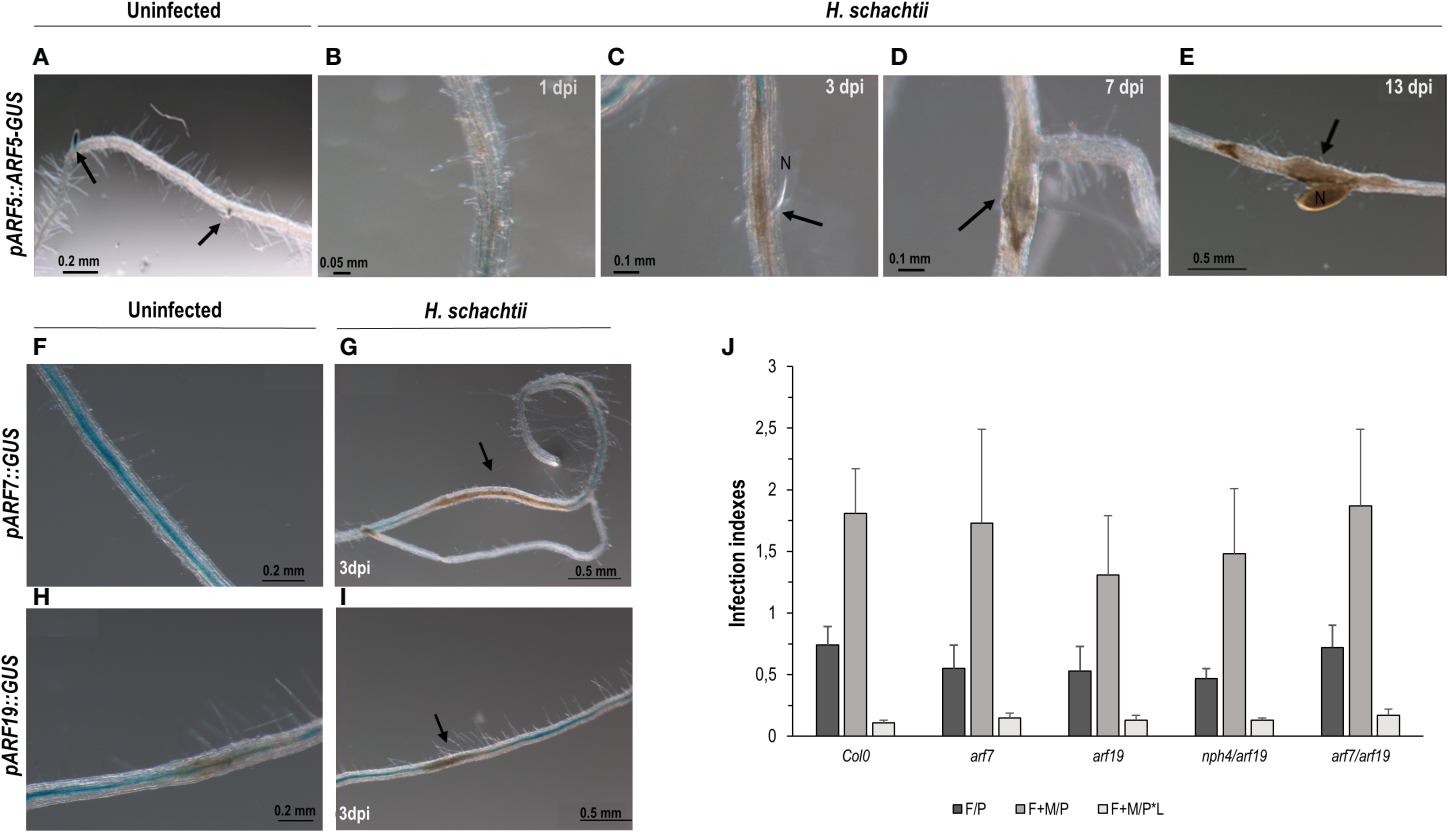
Activation patterns of auxin response factors crucial during lateral root and gall formation (*pARF5::ARF5-GUS*, *pARF19::GUS*, *pARF7::GUS*) in Arabidopsis roots infected with *Heterodera schachtii*. Expression in the root tips of uninfected tissue used as positive control (**A**; Olmo et al., 2020) Arabidopsis roots of *pARF5::ARF5-GUS* infected by *H. schachtii* showed no *GUS* signal in any of the infection stages assessed, either early (1, 3, 7 dpi; **B–D**) or medium late (13 dpi, **E**). The *pARF7::GUS* and *pARF19::GUS* lines showed a GUS signal extended along the roots and sometimes patchy in uninfected tissues **(F, H)** with no obvious defined pattern different in syncytia **(G, I)**. Mutant lines corresponding to ARF7 or ARF19 either single (*arf7*, *arf19*) or double (*arf7/19*, *nph4/arf19*) mutants, showed no significant differences either in the number of females per plant (F/P), or females + males per plant (F + M/P), or the number of females + males per plant normalized to the root length (F + M/P ∗ L; **J**; Student’s t-test; p <0.05), named as infection indexes in the Y axis. Scale bars as indicated. At least n ≥40 independent plants were assayed for GUS per independent line and for the infection parameters of the *arf7*, *arf9*, *arf7/19*, and *nph4/arf19* mutant lines and its corresponding control Col-0. At least three independent experiments per line were performed.

ARF5/7/19 are upstream regulators of *LBD16* and *GATA23* (Okushima et al., 2007; De Rybel et al., 2010); ARF5 is also a putative upstream regulator of *AHP6* (Besnard et al., 2014), all crucial during LR formation. Additionally, ARF5 controls the expression of *miR390* in the root meristem (Dastidar et al., 2019). Likewise, *LBD16*, *GATA23*, *AHP6*, and *miR390* were induced and essential for gall formation (Cabrera et al., 2014a; 2016; Olmo et al., 2020). In this context, we have confirmed that *GATA23* is regulated by auxins in syncytia and that *miR390a* is also partially regulated by auxins in syncytia; therefore, we studied whether ARF5 and/or ARF7/19 might also be involved in the upstream regulation of the expression of those genes during CN infection. The *pARF5::ARF5-GUS* line occasionally showed a pale GUS signal only in cells at the edge of the syncytia (**Figure 4C**; **Supplementary Figure 3**), but no clear signal within the syncytia at any of the infection stages analyzed (1, 3, 7, and 13 dpi; **Figures 4B–E**), although it showed the expected activation pattern in uninfected roots (**Figure 4A**). In addition, the GUS signal in *pARF19::GUS* and *pARF7::GUS* lines could not be localized within the syncytia (**Figures 4G, I**, respectively), but showed a background signal along the roots similar to that of the control non-infected roots or in the syncytia neighboring cells (**Figures 4F–H**). Accordingly, differences in the infection indexes, i.e., the number of females per plant or females + males per plant, or females + males per root length, were not significant between the control Col-0 line and the *arf7*, *arf19*, *arf7/19*, and *nph4(arf7)* mutants (**Figure 4J**). Therefore, no significant differences in the investigated single and double loss of function mutants for both ARF genes were found.

### Distinctive arrangements of AuxRes in the proximal promoter regions correlate with their activation during the root-knot and cyst nematode infections

Interestingly, from the data obtained major differences were observed between the promoter activation of plant auxin-regulated genes by CNs and RKNs, but there were also some similarities (**Figures 1**–**4**; Cabrera et al., 2014a; 2016). We, therefore, aimed to analyze the type of AuxRe sequences present in the proximal promoter regions of *LBD16*, *GATA23*, *AHP6*, and *miR390*. This was to get a better understanding of their regulation after infection with CNs and RKNs. We identified several canonical and non-canonical putative AuxRe *cis*-elements with different rearrangements following the classification of Cherenkov et al. (2018); **Supplementary Figure 2**, **Supplementary Table 1**). Interestingly, the promoters of genes that were not induced after CN infection but activated in galls as well as during LR formation and/or in the root meristem harbor only one or two canonical AuxRe elements (TGTCTC; 2XTGTGGG; *pLBD16* and *pAHP6*, respectively). However, in those promoters activated by *H. schachtii*, i.e., *pGATA23* and different versions and deletions of the *pmiR390a*, several AuxRe elements that correspond to hexamers potentially bound by bHLH and/or bZIP transcription factors were identified, and, in some of them, no canonical AuxRe elements were present (**Supplementary Figure 2**; **Supplementary Table 1**). It is known that ARFs can heterodimerize with other transcription factors, such as members of the bHLH family (Varaud et al., 2011; Oh et al., 2014), among others. In addition, bZIP-binding sites mediate auxin responses but are coupled to AuxREs and enhance auxin-mediated transcription of the *GH3* gene in an auxin concentration-dependent manner (Ulmasov et al., 1995; Weiste and Dröge-Laser, 2014). Then, we looked at members of the bHLH and bZIP families differentially expressed in the transcriptomes of galls, micro dissected GCs and microaspirated syncytia in Arabidopsis, available in the database NEMATIC (Cabrera et al., 2014b). Among the upregulated genes, only one bHLH (*BEE2*; **Supplementary Table 2**) was found in GCs and three (AT1G05710, AT3G07340, and AT2G40200) in galls at 3 dpi, but in syncytia, 11 members of the bHLH family were upregulated (**Supplementary Table 2**). Similarly, only one member of the bZIP family (*POSF21*; **Supplementary Table 2**) was upregulated in galls at 7 dpi, but in syncytia, two bZIP members were upregulated, *BZIP9* and *POSF21* (**Supplementary Table 2**).

**Figure 5:**
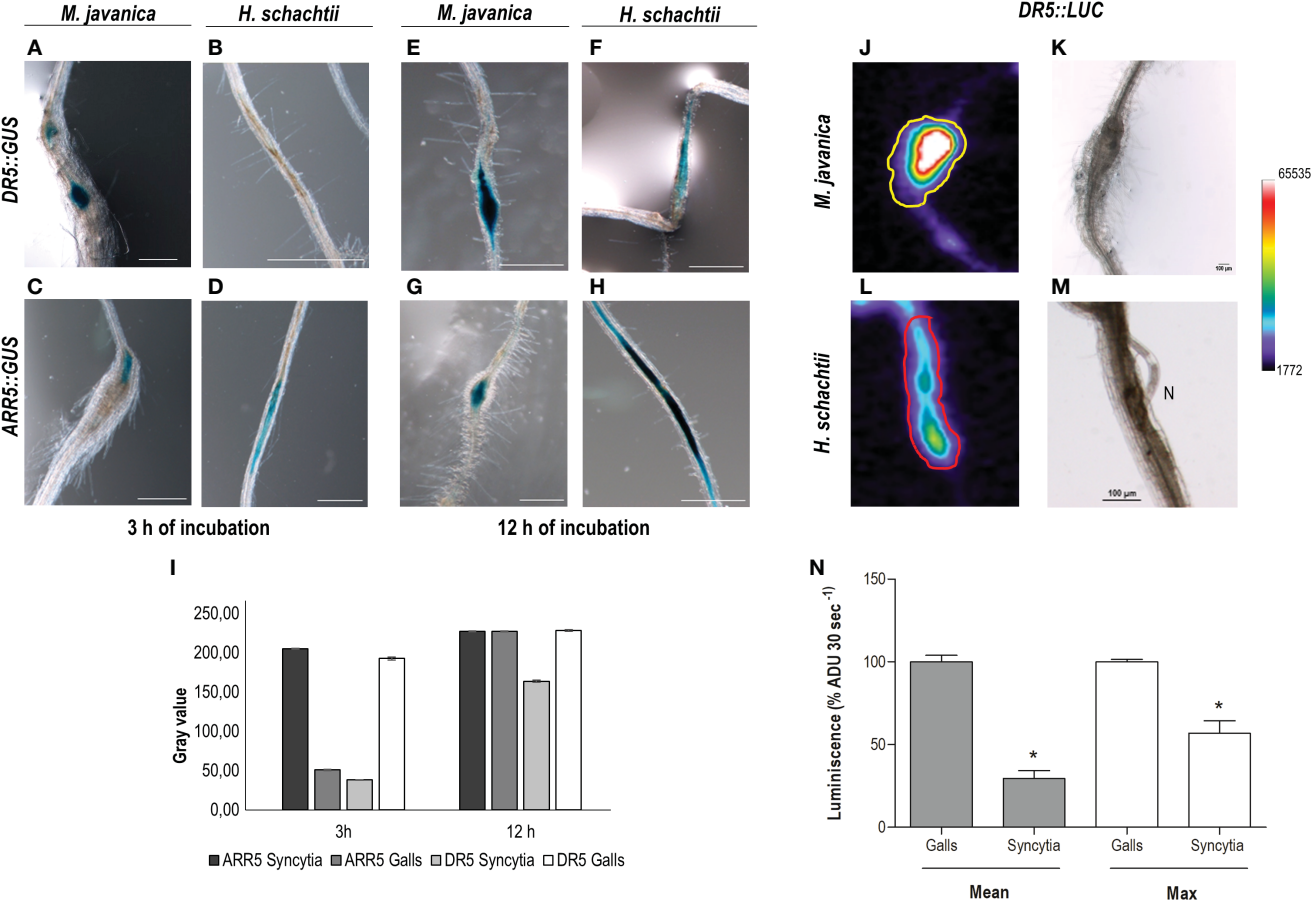
The auxin signaling pathway is more active in RKNs that in CN infection sites, while CN infection sites present a stronger cytokinin signaling pathway at early infection stages. Arabidopsis reporter lines *DR5::GUS, DR5::Luciferase (DR5::LUC)* and *ARR5::GUS* were inoculated with *M. javanica* **(A, C, E, G, J, K)** and with *H. schachtii* **(B, D, F, H, L, M)**, and at 3–4 days post infection (dpi) GUS or LUC were assayed. *DR5::GUS* signal in RKNs **(A, E)** and in CN infection sites **(B, F)** 3 and 12 h after incubation in the GUS reaction. GUS-staining of the *ARR5::GUS* 3 and 12 h after incubation in the GUS reaction in CN **(D, H)** and RKN **(C, G)** infection sites. Semiquantification of GUS histochemical staining in both infection sites of *DR5::GUS* and *ARR5::GUS* lines, after 3 or 12 h incubation (n ≥15 for each RKN or CN infection sites; **(I)**. Scale bars: 500 µm **(A–H)**. At least three independent experiments per independent line assayed were performed. *DR5::LUC* images of RKNs and CNs and infection sites (**J, L**, respectively) and their corresponding transmission images **(K, M)**. Quantification of the luminescence (average signal per pixel in defined Regions of Interest (ROIs), defined in **Supplementary Figure 4**, relative to that of galls, **N**) (N ≥15 CN and RKN infection sites; p <0.05). *Asterisk, significant differences; p <0.05; Student’s t-test). ADU, analogical digital units. Pseudocoloring scale is indicated. Two independent experiments were performed.

It is well described that the *DR5::GUS* or *DR5:.GFP* reporter lines used to indicate that auxin response pathways (mainly IAA-mediated) are activated in different tissues are also active at CN and RKN feeding sites (Hutangura et al., 1999; Karczmarek et al., 2004; Absmanner et al., 2013; Cabrera et al., 2014a; Olmo et al., 2020). Interestingly, DR5 carries a highly active synthetic promoter driving GUS consisting of a heptamer of a direct repeat of the canonical AuxRe element (TGTCTC; Ulmasov et al., 1997), also present in the *LBD16* proximal promoter sequence (**Supplementary Figure 2**). A detailed study following the accumulation of the GUS product in *DR5::GUS* at 3 and 12 h after incubation with the reactive X-gluc (see *Materials and methods*) together with a semi-quantification of GUS histochemical staining in RKN and CN infection sites (3–4 dpi) showed that the GUS signal in the CN infection sites (3–4 dpi) after 3 h of incubation was almost 5-fold lower than in galls (**Figures 5A, B, I**). After 12 h of incubation, although the signal in CN infection sites increased with respect to 3 h incubation, it was still lower than in galls (1.5-fold lower; **Figures 5E, F, I**), what suggests that the DR5 promoter, even carrying a redundant synthetic arrangement of AuxRe, is less active in syncytia than in galls. In contrast, the *ARR5::GUS* line, which uses the ARR5 promoter as a cytokinin-signaling marker (D'Agostino et al., 2000), showed a stronger signal in CN infection sites 3 h after incubation (4-fold) than in galls (**Figures 5C, D, I**), although 12 h after incubation it reached a saturated maximum in both infection sites (**Figures 5G–I**). To use an independent measurement technique, we assayed the same *DR5* promoter fused to another reporter gene encoding *Luciferase* (LUC), *DR5::LUC*, which produces bioluminescence, and analyzed differences in expression in both RKN and CN infection sites. The luminescence produced was measured as described in *Materials and methods*. The data indicated that the signal was stronger in the RKN than in the CN infection sites (p <0.05); using both parameters that is the quantification of the average intensity of the Regions of Interest (ROIs) or the maximum value, showed the same tendency (3.4 and 1.8-fold-change, respectively). All together these results strongly suggest that the auxin responses are enhanced in early developed galls as compared to the syncytia, whereas the cytokinin responses are more pronounced in early developed syncytia as compared to galls, although both signaling responses co-exist in both infection sites.

Taking advantage of the Arabidopsis transcriptomes of the cyst and root-nematode feeding sites available in NEMATIC (Cabrera et al., 2014b), we identified the transcriptional patterns in either galls/GCs or syncytia of 430 genes upregulated after auxin (IAA) treatment as described in Nemhauser et al. (2006). In microaspirated syncytia, 61 out of 430 IAA-induced genes were upregulated, and in microdissected GCs, 20. If we consider the total number of upregulated genes in each differential transcriptome analysed, the highest proportion of IAA-upregulated genes was represented in micro-dissected GCs (6.4%; **Supplementary Table 3**). In contrast, the proportion of upregulated IAA-regulated genes were low in syncytia (1.5%; **Supplementary Table 3**). Interestingly, the number of common upregulated genes induced by IAA between both types of feeding sites was strikingly low, with only two genes in common between syncytia and GCs (**Supplementary Table 4**). We made a similar comparison with those up-regulated auxin-related genes classified in MAPMAN (Usadel et al., 2009), including those related to auxin metabolism and auxin response factors, there were a total of 208 genes. The percentage of upregulated IAA-related genes was also lower in the syncytia transcriptome than in that of GCs (0.49% and 1.29%, respectively), and no common upregulated genes were identified (**Supplementary Tables 3**, **4**). These results indicate that although there is an active auxin signaling response at both feeding sites, just a few auxin-regulated genes are common between both types of nematode feeding sites.

## Discussion

Feeding site formation by CNs is essentially different from that of RKNs. Yet, both feeding sites show an auxin maximum pertinent to feeding site formation (reviewed in Oosterbeek et al., 2021). However, molecular divergences and similarities between the formation of both feeding sites regarding auxin-responsive genes are not yet well described. In this respect, we studied genes induced by auxins and are crucial during gall formation and central to LR development (Cabrera et al., 2014a; Cabrera et al., 2016; Olmo et al., 2020) during the Arabidopsis–CN interaction.

We analyzed the activation of three gene promoters (*GATA23*, *AHP6*, and *miR390*) regulated by auxins in roots that are all essential for gall formation (Cabrera et al., 2016; Olmo et al., 2020) during *H. schachtii* interaction with Arabidopsis. Interestingly, only two (*GATA23* and *miR390*) of the three genes were activated after *H. schachtii* infection. In contrast, all of them were active in galls (**Figures 1**–**3**; Cabrera et al., 2016; Olmo et al., 2020). We confirmed that *pGATA23* and *pmiR390a* respond to auxin signaling in RKN and CN infection sites, as treatment with an antagonist of IAA, PEO-IAA, suppressed either fully (*pGATA23::GUS*; **Figure 1**) or partially (*pmiR390a::GUS*; **Figure 2**) the GUS signaling in both gall and CN infection sites. Likewise, both genes were regulated by auxins during LR formation (De Rybel et al., 2010; Dastidar et al., 2019). GATA23 is involved in the very first stages of LR formation, i.e., in the specification of LR founder cells and during the first divisions of LR primordia (**Figure 1**; De Rybel et al., 2010; Goh et al., 2012), whereas *miR390* is involved in the primordia progression for LR development but not in the initiation process (Dastidar et al., 2019). However, none of them showed a significant functional impact during the infection or establishment of *H. schachtii*, as the loss of function lines for either GATA23 (*GATA23-RNAi*) or *miR390a* (*mir390a-2*) showed no significant impairment in CN infection (**Figures 1**, **3**). We also confirmed that an AuxRe element described in the proximal promoter region of *pmiR390a*, responsible for *miR390a* expression in the root meristem (Dastidar et al., 2019), contributes partially to the activation of *pmiR390a* during CNs and RKNs infection, as either a deletion promoter line of 519 bp (*pMIR390a-519::GUS*) that does not include the AuxRE (CCGACA) element or a deletion itself of the AuxRe (*pMIR390a-555ΔARE::GUS*) were still active in both feeding sites at early and medium-late infection stages. Yet, the proportion of GUS-stained RKNs and CNs infection sites decreased considerably compared to the full promoter (*pmiR390a*) and to a 555 bp deletion that included the AuxRe element (*pMIR390a-555::GUS*; **Figures 2**, **3**). In contrast, *AHP6*, a gene upregulated in galls (Olmo et al., 2020) and induced by an auxin maximum in the protoxylem and during LRP formation (Bishopp et al., 2011; Moreira et al., 2013), was not active at any stage of CN infection (**Figure 1**). Interestingly, AHP6 acts as a negative regulator of cytokinin signaling, playing a key role in the auxin/cytokinin interplay during LR formation (Bishopp et al., 2011; Moreira et al., 2013). The described function of AHP6 is somehow in accordance with the lower cytokinin responsiveness of galls but with the stronger cytokinin response of CN infection sites, measured by *ARR5::GUS* (**Figure 5**; D'Agostino et al., 2000). In this way, AHP6 might act as a negative regulator of the cytokinin response, thus lowering the cytokinin response in galls (Olmo et al., 2020), but not at CN infection sites (**Figure 5**). In agreement with this, Arabidopsis lines with reduced cytokinin sensitivity showed reduced susceptibility to *H. schachtii* infection, indicating that cytokinin signaling is essential for CN establishment (Shanks et al., 2016). Furthermore, the lack of activation of *AHP6* in syncytia could also be in accordance with the lower auxin response detected in CN infection sites as compared to galls measured by the activation of the *DR5::GUS* and *DR5::LUC* auxin response sensors (**Figure 5**).

The activity of *DR5::GUS* and *DR5::LUC* carrying 7× canonical AuxRe elements (TGTCTC) indicates clearly that there is auxin-responsive gene transcription in syncytia (**Figure 5**), which agrees with former reports (Karczmarek et al., 2004; Cabrera et al., 2014a; Olmo et al., 2020). Further indication of active auxin-response gene transcription in early stages of syncytia formation is that treatment with PEO-IAA, an auxin signaling inhibitor, abolished the activation of *pGATA23* in CN infection sites and partially suppressed that of *pmiR390a*. Interestingly, from those genes crucial for gall and LR formation that are regulated by auxins investigated to date, only the promoters of genes carrying AuxRe elements overlapping other cis-elements (bZIP and/or bHLHs binding motifs), i.e., *pGATA23* and *pmiR390a*, were also activated by CNs (**Figures 1**–**3**; **Supplementary Figure 2**; **Supplementary Table 1**). In contrast, the promoters of *pLBD16* and *pAHP6* carrying canonical AuxRes, i.e., one repeat of the DR5 AuxRe motif (TGTCTC) and two similar canonical AuxRe (2× TGTGGG; **Supplementary Figure 2**; **Supplementary Table 1**), respectively, were not induced after CN infection (**Figure 1**; Cabrera et al., 2014b), but they were strongly activated during gall formation (Cabrera et al., 2014a; Olmo et al., 2020, respectively). In this respect, the GUS signal detected in CN infection sites in the *DR5::GUS* and *DR5::LUC* lines, carrying 7× canonical AuxRe motifs (TGTCTC identical to the AuxRe in the *LBD16* promoter), might be due to the high redundancy of the AuxRe present in this construct (7 × AuxRe), which could strongly promote the binding of ARFs, as the DR5 element represents an exceptionally active AuxRe compared with natural composite AuxRes containing the TGTCTC element (Liu et al., 1994; Ulmasov et al., 1995; Ulmasov et al., 1997). Yet, the same ARF-binding site of the native soybean *GH3* promoter that was used as a reference for the *DR5::GUS* line construct was first described as part of a composite auxin response element that binds a bZIP transcription factor (Ulmasov et al., 1995; Liu et al., 1997). Furthermore, bZIP-binding sites are not sufficient to mediate the auxin response themselves, but they couple to AuxRes to enhance the auxin-mediated transcription of *GH3* in an auxin concentration-dependent manner (Ulmasov et al., 1995). In this respect, natural promoters, as is the case for *pGATA23* and *pmiR390a*, might need the participation of ARFs and other cofactors, as for example bZIPs and/or bHLHs, for their activation during CN infection, as overlapping of bZIP and/or bHLH with ARF binding sites was identified in those promoters, but not in the promoters of *LBD16* or *AHP6* (**Supplementary Figure 2**). In agreement, several genes encoding bZIP and bHLH transcription factors were activated in syncytia transcriptomes but very few in GC or gall transcriptomes at early infection stages (**Supplementary Table 2**). Hence, the strong activation of *pLBD16* (Cabrera et al., 2014a) and *pAHP6* (Olmo et al., 2020) described in galls/GCs suggests that contrary to CN infection sites, the presence of canonical AuxRe is sufficient for a strong auxin response in the early stages of gall development. Accordingly, the activity of the *DR5* promoter was stronger in RKNs than in CN infection sites (**Figure 5**).

In agreement with the contrasted differences in the transcriptional regulation of auxin-responsive genes between CNs and RKNs/LRs (**Figures 1**–**3**; **Supplementary Tables 3**, **4**), the promoters of three of the main auxin-related transcription factors involved in LR formation, ARF5, 7, and 19, that act through IAA14/ARF7-ARF19 and IAA12/ARF5 signaling to activate *LBD16* and *GATA23*, respectively, in LRs (Fukaki et al., 2005; De Smet et al., 2010), were not active in syncytia (**Figure 4**). These results are in line with data from *ARF7* and *19* promoter-GFP fusions that showed high expression mainly in the syncytia and neighboring cells (Hewezi et al., 2014). However, the activation pattern of *pARF5::GFP* (Hewezi et al., 2014) did not concur with that of *pARF5::ARF5-GUS* at early infection stages (**Figure 4**). Differences in the promoter regions and the reporter genes used, as well as the fact that *pARF5::ARF5-GUS* is a translational fusion to GUS that measures ARF5-GUS accumulation, whereas *pARF5::GFP* is a transcriptional fusion that measures ARF5 promoter activity, might cause the observed differences. However, the same *pARF5::ARF5-GUS* line, with no evident expression in CN infection sites in this study (**Figure 4**), was strongly upregulated and functional in galls (Olmo et al., 2020). Accordingly, only *ARF3*, *4*, and *6* transcripts accumulated in micro-aspirated syncytia (**Supplementary Table 1**; Szakasits et al., 2009) and *ARF9* in the CN–Arabidopsis interaction (Oosterbeek et al., 2021), but not *ARF5*. Therefore, it is reasonable to think that other ARFs different from ARF5/7/19 should be involved in *GATA23* or *miR390a* promoter activation in syncytia through the AuxRe elements present in their proximal promoters. Nevertheless, it is quite feasible that transduction cascades mediated by Aux/IAA proteins, known upstream regulators and repressors of ARFs, are active in CN infection sites, as they are in galls (IAA12, IAA14, and IAA28; Olmo et al., 2020). Some of the lines of evidence are that several Aux/IAA genes are upregulated in CN feeding sites (Oosterbeek et al., 2021), a dominant mutant Arabidopsis line of IAA14 (*slr*) was more resistant to the infection of *H. schachtii* (Grunewald et al., 2008), and that the effector protein 10A07 of *H. schachtii* physically interacts with Aux/IAA16 (IAA16), which concurs with changes in CN susceptibility in IAA16 and IAA7 loss of function mutant Arabidopsis lines (Hewezi et al., 2015). Interestingly, and in line with the partial activation by auxins of *pmiR390a::GUS* in syncytia (**Figures 2**, **3**), *WRKY23*, an auxin-regulated gene in uninfected plants through the IAA14 pathway, was regulated by signals other than auxins in syncytia (Grunewald et al., 2008).

**Figure 6:**
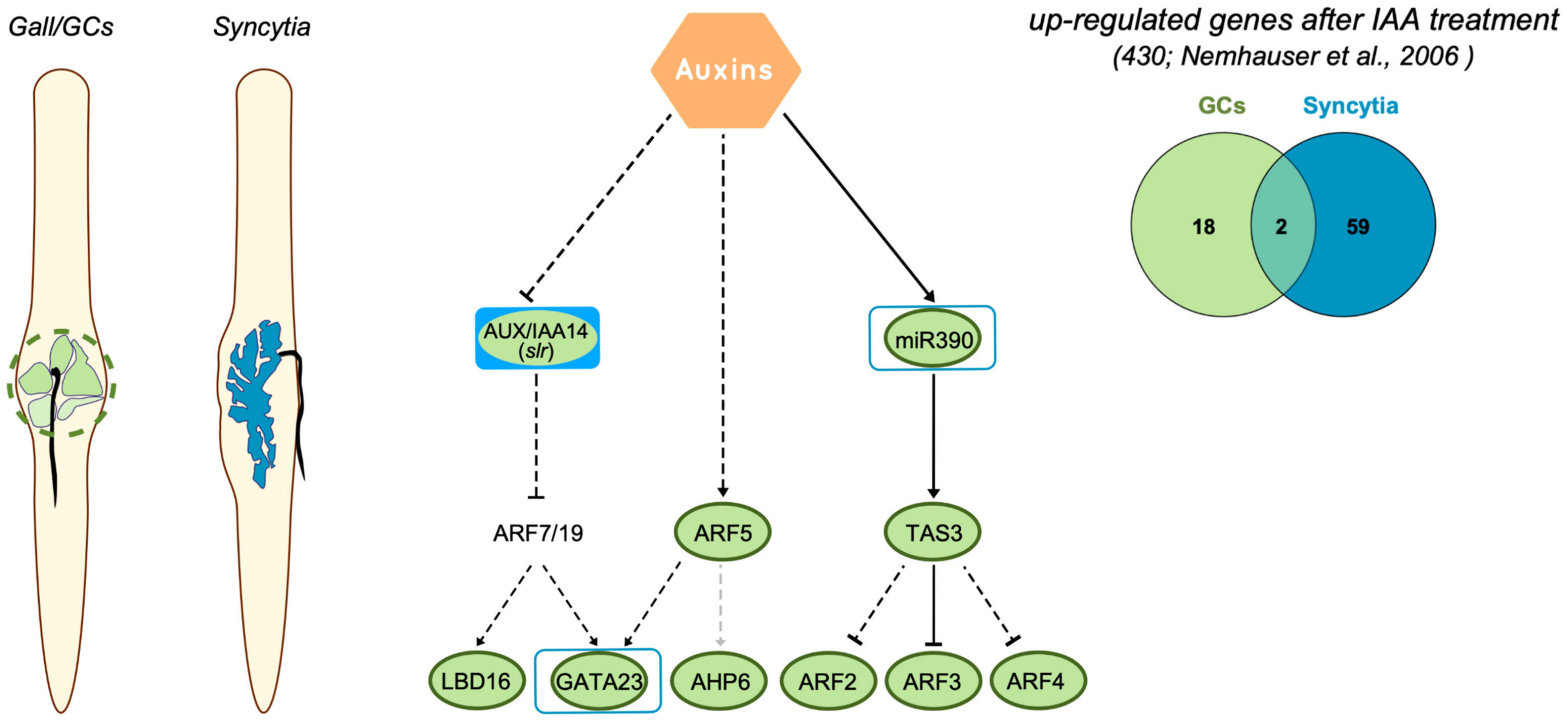
Regulation of auxin-responsive genes crucial for LR formation in CN and RKN infection sites of Arabidopsis. Right panel, metanalysis of genes induced after IAA treatment in Arabidopsis (Nemhauser et al., 2006) that are also upregulated in giant cells (GCs) formed by *M. javanica* and microaspirated syncytia. The number of common genes between GCs *versus* syncytia is quite low. Left panel, representation of roots infected with root-knot nematodes and cyst nematodes, in green the GCs, in blue the syncytia. Central panel, diagram representing a pathway modified from Dastidar et al. (2012) and based on Besnard et al. (2014) with main genes regulated by auxins in LR formation and during galls and syncytia development. Dashed black lines indicate that regulation is confirmed during LR formation, but not yet in the root-nematode interaction, dashed grey lines indicate not yet confirmed for LR. The expression of most of the genes in galls was obtained from (Cabrera et al., 2014b; Olmo et al., 2020 and this paper). Those forms with solid green or blue inside indicate that the corresponding loss of function line for the gene showed a resistant phenotype after root-knot nematode or cyst nematode infection, respectively. Green lines indicate induction in RKN infection sites, blue lines indicate induction in CN infection sites.

In conclusion, auxin is a relevant hormone for the morphogenesis of CN and RKN feeding sites (2015; Goverse et al., 2000; Cabrera et al., 2014a; Olmo et al., 2020; Oosterbeek et al., 2021). It is clearly established that with the aid of imbalances in auxin transport promoted by differential expression and localization of a specific combination of PIN-FORMED (PIN; efflux auxin carriers) and AUX1/LAX family proteins (influx carriers) in both syncytia (Grunewald et al., 2009; Lee et al., 2011) and GCs (Kyndt et al., 2016), and perhaps with the contribution of auxin-like compounds identified in nematode secretions (De Meutter et al., 2005; Goverse and Bird, 2011), as well as with the manipulation of catabolic and synthetic pathways (reviewed in Oosterbeek et al., 2021), an auxin maximum is built that triggers feeding cell formation. However, the transcriptional responses to auxins are diverse and complex, as many of the members of the signaling transduction pathways belong to large families (e.g., 23 ARF proteins were identified in Arabidopsis) (Rademacher et al., 2011). Additionally, ARFs form homo and heterodimers with other transcription factors, such as bHLHs, or indirectly with bZIPs, among others (Ulmasov et al., 1997; Guilfoyle et al., 1998; Vernoux et al., 2011; Boer et al., 2014), activating genes through AuxRe elements that sometimes are part of a composite recognized by transcription factors other than ARFs (Ulmasov et al., 1995; Cherenkov et al., 2018). This diversity of auxin signaling pathways may explain why the transcriptional responses governed by auxins show contrasted differences between the CN and RKN feeding sites, at least at early medium stages of infection. Yet, despite sharing common upstream auxin regulators (Aux/IAAs, e.g., IAA14), crucial either for CN or for RKN infection (Grunewald et al., 2008; Olmo et al., 2020), the two types of feeding sites do not share downstream regulators such as ARF5 or transcription factors downstream of ARFs (*LBD16* or *AHP6*) that are not activated during the CN–Arabidopsis interaction but highly expressed and crucial for LR and gall/GC formation (**Figures 1**, **6**; Cabrera et al., 2014a; Olmo et al., 2020). It also could explain why the number of common auxin-regulated genes between GCs and syncytia was strikingly low as compared to the number of auxin-responsive genes in each of the individual transcriptomes of the two types of plant–nematode interaction (CNs and RKNs) (**Figure 6**; **Supplementary Tables 3**, **4**). Furthermore, it would also explain that *GATA23* or *miR390a* share similar promoter activation responses in both RKN and CN infection sites (**Figures 1**–**3**, **6**; Cabrera et al., 2016; Olmo et al., 2020), even though both GATA23 and *miR390a* seem quite dispensable for CN establishment (**Figures 1**, **3**), but crucial for gall formation (Cabrera et al., 2016; Olmo et al., 2020). However, another scenario might also be possible, as other types of endogenous auxins, as suggested by Oosterbeek et al. (2021), which are not well characterized yet, may trigger additional transduction pathways that are still unknown and divergent in both nematode feeding sites. In addition, the identification of RKN and CN effectors such as the CLE-like peptides with high similarity to those found in plants (reviewed in Mitchum and Liu, 2022) that show complex signaling cascades coordinated with hormones, e.g., auxins, to regulate plant development (Wang et al., 2016), is still an open field of research regarding their impact on NF formation. Further research will elucidate this divergent and complex regulation mediated by auxins at both feeding sites.

## Data availability statement

The datasets presented in this study can be found in online repositories. The names of the repository/repositories and accession number(s) can be found in the article/**Supplementary Material**.

## Author contributions

PA-U was involved in most of the experiments and in the in silico comparisons, as well as in the writing of the manuscript. VR-F in some experiments and in silico comparisons. JD-F, ACS, RO, FED-M, AM-G, AG-R and JC performed some of the experiments presented. MAM-R guided and participated in the experiments regarding DR5::LUC promoter activity quantification. CE coordinated and designed the experiments and participated in the writing of the manuscript, and CF participated in the correction of the manuscript. All the authors contributed to the critical reading of the manuscript.
