# Supplementary material for: Divergent regulation of auxin responsive genes in root-knot and cyst nematodes feeding sites formed in Arabidopsis
Source: Front Plant Sci. 2023 Feb 15;14:1024815. doi: 10.3389/fpls.2023.1024815 (PMC9976713; doi:10.3389/fpls.2023.1024815)
Supplement: Supplementary file 1 [file DataSheet_1.pdf]

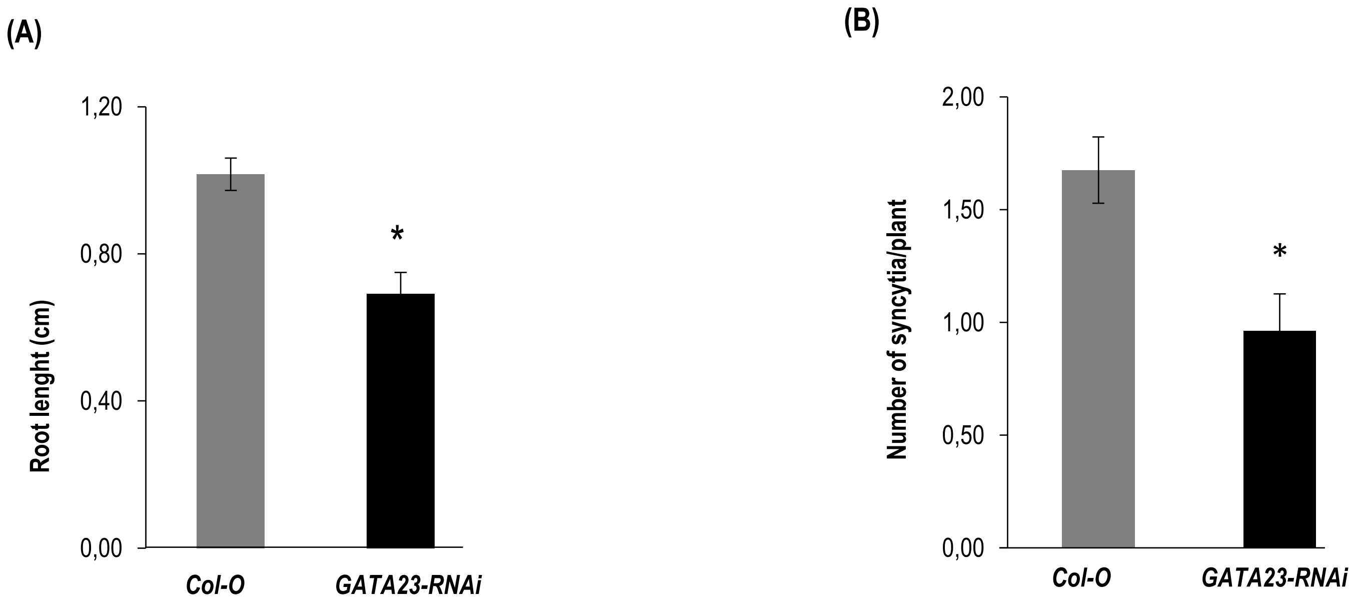

Fig. S1. Root length of seedlings before nematode inoculation of a RNA interference line, *GATA23-RNAi* (De Rybel et al., 2010*)* compared to Col-O (A). Number of syncytia per plant after inoculation with *H. schachtii* in the *GATA23-RNAi* line and Col-O (B). *Asterisk, significant differences ($p<0.05$; student´s t-test).

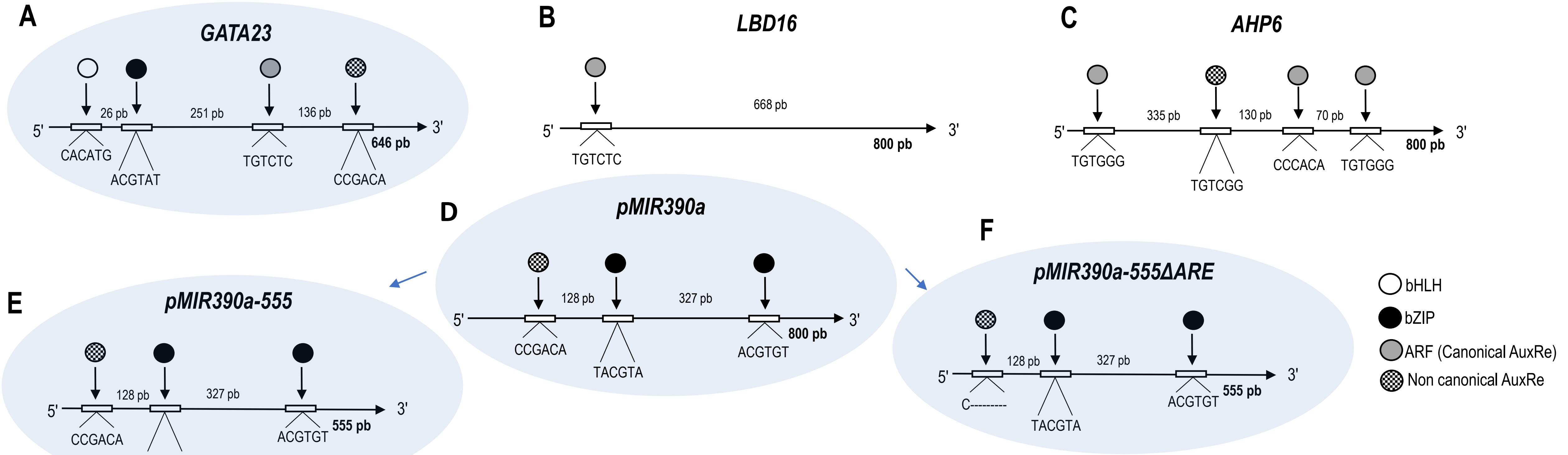

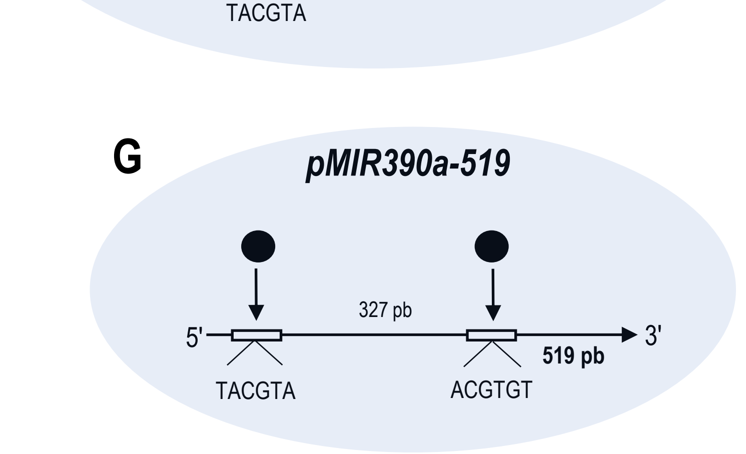

Fig. S2. *AuxRe* elements identified in several promoters of genes activated and central during galls and lateral root (LR) formation. A list of canonical AuxRe (Table S1) in direct and inverted orientation, as well as a list of AuxRe defined in Cherenkov et al. (2018) overlapping bZIP and bHLH transcription factor families binding sites (Table S1) and a non-canonical, AuxRe defined in Dastidar et al. (2019), were localized within the proximal promoter regions (800 bps from the transcription start point) of *GATA23* (A) *LBD16* (B), *AHP6* (C) and *miR390a* (D). Three deletions of the *pmiR390a*, one including a non-canonical AuxRe (*pmiR390a-555*; Dastidar et al. (2019), (E), and two lacking either a 36 bp region that includes the non-canonical AuxRe (*pmiR390a-519*, G) or 5 nucleotides of the non-canonical AuxRE (*pmiR390a-555ΔARE,* F) are included. Those AuxRe identified within the promoters are indicated, as well as their sequences. In light blue, those promoters activated during the cyst nematode interaction (*H. schachtii*) as well as during gall and LR formation. In white, those only induced in galls and during LR formation. Black circle, AuxRe overlapping bZIP transcription factors, white circle, AuxRe overlapping bHLH transcription factors, grey circle, canonical AuxRe, circle filled with lines, noncanonical AuxRe.

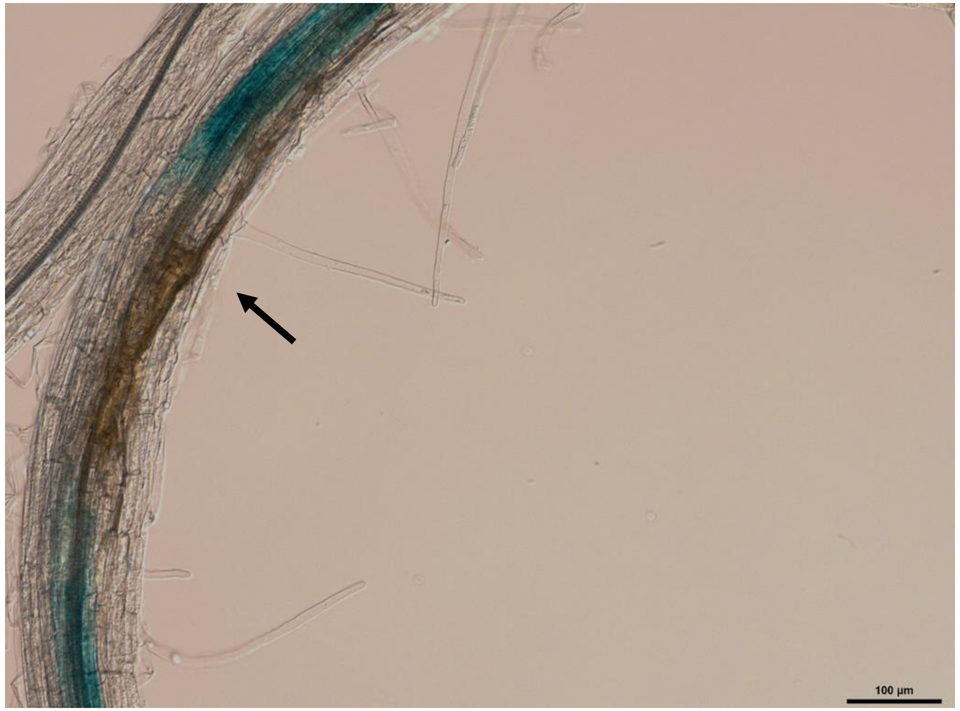

Fig. S3. Representative Arabidopsis root segment of the *pARF5::ARF5-GUS* line showing some GUS signal in the cells surrounding the synctytia (black arrow) of *H. schachtii* at 7dpi. This signal is occasionally observed in some infected roots.

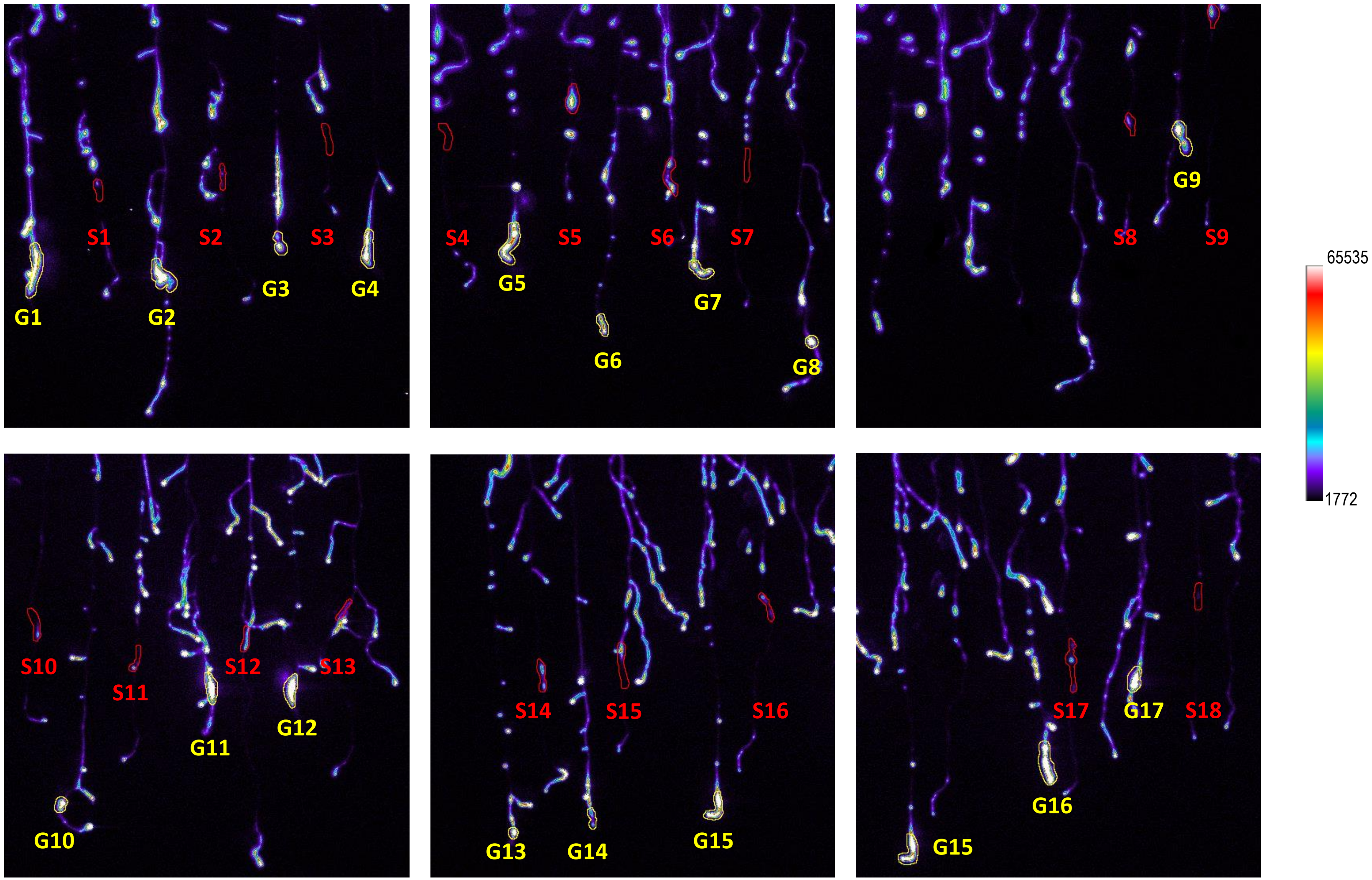

Fig. S4. **Luciferase images of plants infected with *M. javanica* or *H. schachtii* used for quantification of luminescence.** Pseudocoloring scale is indicated. The regions of interest (ROIs) including the CNs, in red (S1-S18), and RKNs infection sites, in yellow (G1-G17), are indicated.

Table. S1. AuxREs defined in Cherenkov et al. (2018) identified in the proximal promoter regions (800 bps from the transcription start point) of *GATA23, LBD16*, *AHP6* and *miR390a,* including two deletions of 519 and 555 bps of *pmiR390a* and *555ΔARE.* A non-canonical *AuxRe* functional in the promoter region of *miR390a,* is also included (Dastidar et al., 2019).

	Canonical AuxRe binding sites	AUxRe overlaping bHLH sites	AuxRe overlaping bZIP sites	Non-canonical AuxRE
GATA23 **(At5G26930)**	TGTCTC	CACATG	ACGTAT	CCGACA
LBD16 **(At2G42430)**	TGTCTC	-	-	-
AHP6 **(At1G80100)**	TGTGGG<br>TGTGGG<br>CCCACA	-	-	TGTCGG
pmiR390 **(At2G38325)**	-	-	TACGTA<br>ACGTGT	CCGACA
pmiR390-519	-	-	TACGTA<br>ACGTGT	-
pmiR390-555	-	-	TACGTA<br>ACGTGT	CCGACA
pmiR390-555ΔARE	-	-	TACGTA<br>ACGTGT	-

Table S2. Identification of genes encoding transcriptions factors from the Auxin responsive factor (ARFs), Basic Leucine Zipper Domain (bZIP) and Basic Helix-Loop-Helix (bHLH) family members up regulated either in micro dissected giant cells, galls or microaspirated syncytia performed from the lists available in NEMATIC (Cabrera et al., 2014b). ID of the genes, a brief description of the predicted protein product from TAIR, and the transcription factor family are indicated in each column. Up, up regulated, -, non-differentially expressed.

Abreviated name	ID	Gene description	GC 3dpi	Gall 3 dpi	Gall 7 dpi	Sync 5+15 dpi
			Transcriptional regulation/ARF			
ARF19	AT1G19220	Auxin responsive factor that together with *ARF7*, is involved in the response to ethylene	UP	-	-	-
ARF5	AT1G19850	Auxin responsive factor mediating embryo axis formation and vascular development	UP	UP	-	-
ARF4	AT5G60450	Auxin responsive factor with redundant function with *ETT(ARF3)* in specifying abaxial cell identity	-	-	-	UP
ARF3	AT2G33860	Auxin responsive factor with pleiotropic effects on Arabidopsis flower development patterning defects in the gynoecium	-	-	-	UP
ARF6	AT1G30330	Auxin responsive factor that acts redundantly with *ARF8* to control stamen elongation and flower maturation	-	-	-	UP
			Transcriptional regulation/Bzip			
POSF21	AT2G31370	Basic-leucine zipper (bZIP) transcription factor family protein	-	-	UP	UP
BZIP9	AT5G24800	Encodes bZIP protein BZO2H2	-	-	-	UP
			Transcriptional regulation/bHLH			
BEE2	AT4G36540	bHLH domain. Encodes the brassinosteroid signaling component BEE2 (BR-ENHANCED EXPRESSION 2). Positively modulates the shade avoidance syndrome in Arabidopsis seedlings	UP	-	-	-
	AT1G05710	Basic helix-loop-helix (bHLH) DNA-binding superfamily protein	-	UP	-	-
CIB3	AT3G07340	Basic helix-loop-helix (bHLH) DNA-binding superfamily protein	-	UP	-	-
	AT2G40200	Basic helix-loop-helix (bHLH) DNA-binding superfamily protein	-	UP	-	-
	AT5G64980	Transcription factor	-	-	-	UP
	AT5G62610	Basic helix-loop-helix (bHLH) DNA-binding superfamily protein	-	-	-	UP
SACL1	AT5G09460	Transcription factor bHLH143	-	-	-	UP
IBL1	AT4G30410	Sequence-specific DNA binding transcription factor	-	-	-	UP
BHLH60	AT3G57800	Together with *bHLH48* associates with phytochrome interacting factor 7 to regulate hypocotyl elongation	-	-	-	UP
CIL2	AT3G23690	Basic helix-loop-helix (bHLH) DNA-binding superfamily protein	-	-	-	UP
BHLH34	AT3G23210	Basic helix loop helix transcription factor. It can bind GAGA and E-box cis elements	-	-	-	UP
BHLH48	AT2G42300	Together with *bHLH60* associates with phytochrome interacting factor 7 to regulate hypocotyl elongation	-	-	-	UP
BPEP	*AT1G59640*	A basic helix-loop-helix encoding gene (*BIGPETAL, BPE*) involved in the control of petal size	-	-	-	UP
CIB4	*AT1G10120*	Basic helix-loop-helix (bHLH) DNA-binding superfamily protein	-	-	-	UP
BHLH007	*AT1G03040*	Governs the competence of pericycle cells to initiate lateral root primordium formation	-	-	-	UP

Table S3: Upregulated genes in micro-dissected GCs, galls at different developmental stages and microaspirated syncytia from those up-regulated after IAA treatment (430; Nemhauser et al., 2006) and from those auxin-related genes that belong to the "hormone metabolism.auxin" and "auxin responsive factors" categories from MAPMAN (208; Usadel et al., 2009) and also auxin induced in NEMATIC (Cabrera et al., 2014b). The percentages of genes are calculated respect to the up-regulated genes in each of the transcriptomes analysed.

	number of IAA regulated genes (Nemhauser et al., 2006)	total number of up-regulated genes	Percentage of IAA regulated%
**GCs**	20	310	6,45
**Gall 3dpi**	18	354	5,08
**Gall 7dpi**	11	693	1,59
**Syncytia**	61	3890	1,57

	upregulated auxin metabolism and auxin response factors (Mapman)	total number of up-regulated genes	Percentage of IAA regulated%
**GCs**	4	310	1,29
**Gall 3dpi**	3	354	0,85
**Gall 7dpi**	1	693	0,14
**Syncytia**	18	3890	0,46

Table S4: Common genes upregulated in micro-dissected GC, galls at different developmental stages and microaspirated syncytia from those up-regulated after IAA treatment (430; Nemhauser et al., 2006) and from those auxin-related genes that belong to the "hormone metabolism.auxin" and "auxin responsive factors" categories from MAPMAN (208; Usadel et al., 2009) in NEMATIC (Cabrera et al., 2014b). The percentages of genes are calculated respect to the total number of IAA up-regulated genes (Nemhauser et al., 2006) in each transcriptome

IAA upregulated genes (Nemhauser et al., 2006)	common	Percentage in (GC/Galls)%	Percentage in (syncytium)%
GCs	2	10,00	3,28
Gall 3dpi+ Syncytia	6	33,33	9,84
Gall 7dpi+ Syncytia	3	27,27	4,92

upregulated auxin metabolism and auxin response factors (Mapman)	common	Percentage in(GC/Galls)%	Percentage in (syncytium)%
GCs+syncitia	0	0,00	0,00
Gall 3dpi+ Syncytia	1	33,33	5,56
Gall 7dpi+ Syncytia	0	0,00	0,00

Common genes in RKNs and CNs infection sites

In yellow those present in more than one group

IAA upregulated genes (Nemhauser et al., 2006)

UP:syncytia + GCs

	GCS	GALLS 3DPI	GALLS 7DPI	SYNCYTIA	TAIR DESCRIPTION	CLASSIFICATION MAPMAN
AT2G25790	1,11	No	No	1,30	leucine-rich repeat transmembrane protein kinase, putative | chr2:11000599-11004235 FORWARD	30.2.4 - signalling.receptor kinases.leucine rich repeat IV
AT4G25810	1,08	1,73	No	5,10	XTR6: xyloglucan endotransglycosylase-related protein (XTR6)	10.7 - cell wall.modification

UP: Syncytia + Galls 3dpi

	GCS	GALLS 3DPI	GALLS 7DPI	SYNCYTIA	TAIR DESCRIPTION	
AT1G28400	No	0,95	2,02	3,00	unknown protein | chr1:9972384-9973793 REVERSE	35.1 - not assigned.no ontology
AT2G39350	No	1,37	No	1,90	ABCG1: ABC transporter family protein | chr2:16430085-16432557 REVERSE	34.16 - transport.ABC transporters and multidrug resistance systems
AT2G47260	No	1,40	No	1,90	WRKY23: Encodes a member of WRKY Transcription Factor; Group I. Involved in nematode feeding site establishment.	27.3.32 - RNA.regulation of transcription.WRKY domain transcription factor family
AT4G08950	No	2,13	-0,76	3,80	EXO: Symbols: EXO | EXO (EXORDIUM) | chr4:5740297-5741524 FORWARD	30.1 - signalling.in sugar and nutrient physiology
AT4G25810	1,08	1,73	No	5,10	XTR6: xyloglucan endotransglycosylase-related protein (XTR6)	10.7 - cell wall.modification
AT5G66440	No	1,54	No	1,60	unknown protein | chr5:26530235-26531097 REVERSE	35.2 - not assigned.unknown

UP:Syncytia+ Galls 7dpi

	GCS	GALLS 3DPI	GALLS 7DPI	SYNCYTIA	TAIR DESCRIPTION	
AT1G28400	No	0,95	2,02	3,00	unknown protein | chr1:9972384-9973793 REVERSE	35.1 - not assigned.no ontology
AT3G16310	No	No	0,86	2,40	mitotic phosphoprotein N' end (MPPN) family protein | chr3:5526414-5528602 REVERSE	35.1 - not assigned.no ontology
AT5G19440	No	No	1,15	2,30	similar to Eucalyptus gunnii alcohol dehydrogenase of unknown physiological function (GI:1143445), apple tree, PIR:T16995; NOT a cinnamyl-alcohol	26.11.1 - misc.alcohol dehydrogenases.cinnamyl alcohol dehydrogenase

upregulated auxin metabolism and auxin response factors (Mapman)

UP: Syncytia + Galls 3dpi

	GCS	GALLS 3DPI	GALLS 7DPI	SYNCYTIA	TAIR DESCRIPTION	CLASSIFICATION MAPMAN
at2g36210	No	1,05	No	No	auxin-responsive family protein | chr2:15186040-15186804 REVERSE	17.2.3 - hormone metabolism.IAA Reg.induced-regulated-responsive-activated
at4g27450	No	2,96	No	1,10	unknown protein | chr4:13727484-13728886 REVERSE	17.2.3 - hormone metabolism.IAA Reg.induced-regulated-responsive-activated
at1g19850	4,11	1,79	No	No	MP: Encodes a transcription factor (IAA24) mediating embryo axis formation and vascular development. Similar to AUXIN RESPONSIVE FACTOR 1 (ARF1) shown	27.3.4 - RNA.regulation of transcription.ARF, AuxinbResponse Factor family

Table S5. A list of the plant lines used in this manuscript including references, name and Arabidopsis background

Lines	Ecotype	Reference
pARF5/MP::MP-GUS	Col-0	De Rybel *et al*., 2010
pARF7::GUS	Col-0	Okushima *et al*., 2005
pARF19::GUS	Col-0	Okushima *et al*., 2005
pGATA23:GUS	Col-0	De Rybel *et al*., 2010
pAHP6::GUS	Col-0	Mähönen *et al*., 2006
ARR5::GUS	Col-0	D'Agostino *et al*., 2000
DR5::GUS	Col-0	Ulmasov *et al*., 1997
pmiR390a::GUS	Col-0	Marin *et al*., 2010
pmiR390a-555::GUS	Col-0	Dastidar *et al*., 2019
pmiR390a-519::GUS	Col-0	Dastidar *et al*., 2019
*pmiR390a-555*ΔARE::GUS	Col-0	Dastidar *et al* ., 2019
GATA23-RNAi	Col-0	De Rybel *et al.,* 2010
miR390-a	Col-0	Marin *et al.,* 2010
arf7	Col-0	Harper *et al.,* 2000
arf19	Col-0	Harper *et al.,* 2000
arf7/19	Col-0	Harper *et al.,* 2000
nph4/arf19	Col-0	Harper *et al.,* 2000

References:

Dastidar, M. G., Scarpa, A., Mägele, I., Ruiz-Duarte, P., von Born, P., Bald, L., ... & Maizel, A. 2019. ARF5/MONOPTEROS directly regulates miR390 expression in the *Arabidopsis thaliana* primary root meristem. *Plant direct* **3**(2), e00116.

D'Agostino IB, Deruère J, Kieber JJ. 2000. Characterization of the response of the Arabidopsis response regulator gene family to cytokinin. *Plant Physiology* **124**: 1706-1717.

De Rybel B, Vassileva V, Parizot B, Demeulenaere M, Grunewald W, Audenaert D, Van Campenhout J, Overvoorde P, Jansen L, Vanneste S. 2010. A novel aux/IAA28 signaling cascade activates GATA23-dependent specification of lateral root founder cell identity. *Current Biology* **20**: 1697-1706.

Harper, R. M., Stowe-Evans, E. L., Luesse, D. R., Muto, H., Tatematsu, K., Watahiki, M. K., ... & Liscum, E. 2000. The NPH4 locus encodes the auxin response factor ARF7, a conditional regulator of differential growth in aerial Arabidopsis tissue. *The Plant Cell* **12**(5), 757-770.

Mähönen AP, Bishopp A, Higuchi M, Nieminen KM, Kinoshita K, Tormakangas K, Ikeda Y, Oka A, Kakimoto T, Helariutta Y. 2006. Cytokinin signaling and its inhibitor AHP6 regulate cell fate during vascular development. *Science* **311**: 94-98.

Marin, E., Jouannet, V., Herz, A., Lokerse, A. S., Weijers, D., Vaucheret, H., ... & Maizel, A. 2010. miR390, Arabidopsis TAS3 tasiRNAs, and their AUXIN RESPONSE FACTOR targets define an autoregulatory network quantitatively regulating lateral root growth. *The Plant Cell* **22**(4), 1104-1117.

Okushima, Y., Overvoorde, P. J., Arima, K., Alonso, J. M., Chan, A., Chang, C., ... & Onodera, C. 2005. Functional genomic analysis of the AUXIN RESPONSE FACTOR gene family members in Arabidopsis thaliana: unique and overlapping functions of ARF7 and ARF19. *The Plant Cell* **17**(2), 444-463.

Ulmasov T, Murfett J, Hagen G, Guilfoyle TJ. 1997. Aux/IAA proteins repress expression of reporter genes containing natural and highly active synthetic auxin response elements. *The Plant Cell* **9**: 1963-1971.

Table S6. Statistical analysis, ANOVA followed by post-hoc DUNCAN of the number of GUS- stained infection sites from *H. schachtii* and *M. javanica* among the GUS-reporter *Arabidopsis* lines assayed (*pMIR390a-555::GUS, pMIR390a-519::GUS, pMIR390a-555ΔARE::GUS*). Significant differences were stablished with $p<0.05$.

ANOVA FOLLOWED BY POST HOT DUNCAN

H. schachtii										GROUPS
ANOVA						Duncan[a]				
BLUE SYNCYTIA	Sum of squares	df	Mean square	F	Sig.	Line	N	Subset for alpha = 0.05: 1	2	
Between groups	164,667	2	82,333	6,558	0,031	***pMiR390a-555ΔARE::GUS***	3	3,3333		**a**
Within groups	75,333	6	12,556			***pMiR390a-555::GUS***	3		11,6667	**b**
Total	240,000	8				***pMiR390a-519::GUS***	3		13,0000	**b**

Means for groups in homogeneous subsets are displayed.
a. Uses harmonic mean sample size = 3,000

M. javanica										GROUPS
ANOVA						Duncan[a]				
BLUE GALLS	Sum of squares	df	Mean square	F	Sig.	Line	N	Subset for alpha = 0.05: 1	2	
Between groups	764,222	2	382,111	5,065	0,051	***pMiR390a-555ΔARE::GUS***	3	3,6667		**a**
Within groups	452,667	6	75,444			***pMiR390a-519::GUS***	3	12,0000	12,0000	**a, b**
Total	1216,889	8				***pMiR390a-555::GUS***	3		26,0000	**b**

Means for groups in homogeneous subsets are displayed.
a. Uses harmonic mean sample size = 3,000
